# Exploring market-approved azoles as potential breast cancer therapeutics targeting the VEGFR-2 biotarget

**DOI:** 10.1038/s41598-025-29322-6

**Published:** 2025-12-10

**Authors:** Asmaa M. Atta, Mohamed S. Nafie, Hashim A. Ali, Heba K. Saleh, Mohamed A. Roshdy, Mariam M. Mahmoud, Engy A. Eid, Marwa A. Ahmed, Mario S. Gerges, Ahmed M. Ghoneim, Alaa H. Refaie, Eman E. Attia, Mardi M. Algandaby, Khaled M. Darwish

**Affiliations:** 1 Pharmaceutical Chemistry Department, Faculty of Pharmacy, Badr University in Cairo (BUC), Badr City, 11829 Cairo Egypt; 2 Department of Chemistry, College of Sciences, University of Sharjah, P.O. 27272, Sharjah, United Arab Emirates; 3 Bioinformatics and Functional Genomics Research Group, Research Institute of Sciences and Engineering (RISE), University of Sharjah, 27272, Sharjah, United Arab Emirates; 4 Chemistry Department, Faculty of Science, Suez Canal University, P.O. 41522, Ismailia, Egypt; 5 Faculty of Pharmacy, Badr University in Cairo (BUC), Badr City, 11829 Cairo Egypt; 6 Department of Biological Sciences, Faculty of Science, King Abdulaziz University, P.O. 21589, Jeddah, Saudi Arabia; 7 Department of Medicinal Chemistry, Faculty of Pharmacy, Galala University, P.O. 43713, New Galala, Egypt; 8 Medicinal Chemistry Department, Faculty of Pharmacy, Suez Canal University, P.O. 41522, Ismailia, Egypt

**Keywords:** Azoles, Drug repurposing, VEGFR-2, Molecular modelling, Breast cancer, Animal model, Cancer, Drug discovery

## Abstract

Cancer is a complicated, deadly disease, ranking as the second leading cause of death globally. The World Health Organization (WHO) reported nearly 20 million new cases of cancer in 2022, and this burden is anticipated to be doubled by 2035. Breast cancer is one of the most prevalent malignancies and the leading cause of mortality among women globally. As per the GLOBOCAN 2022 database, breast cancer represented 25% of cancer incidence and around 20% of cancer deaths among women in the Middle East and North Africa region (MENA). Although there have been significant developments in surgical, radiotherapeutic, and immunological strategies, chemotherapy remains the major therapeutic approach for cancer treatment. Therefore, there is an urgent necessity for the evolution of innovative and effective therapies against cancer. Vascular endothelial growth factor receptor-2 (VEGFR-2) is an essential regulator of angiogenesis, significantly influencing cancer proliferation and is considered among the most promising targets for cancer treatment. Notably, azole-based derivatives appeared as a promising pharmacophore in combating various diseases and possessed a significant potential to emerge as effective therapeutic options for cancer treatment. Pacing up the drug discovery process, drug repurposing has emerged as a prospective approach owing to its efficiency in saving both time and cost for proven drug kinetics and safety profile. Herein, molecular modelling simulations-coupled biological testing were applied to screen and investigate the potential inhibitory activity of some market-approved azoles against VEGFR-2 biotarget. The anti-fungal azole itraconazole emerged as a promising agent targeting VEGFR-2 to halt angiogenesis and tumour growth, warranting further preclinical and clinical investigations.

**Supplementary Information:**

The online version contains supplementary material available at 10.1038/s41598-025-29322-6.

## Introduction

Cancer, a complex and multifaceted disease, represents a serious challenge to world health. The disease is distinguished by uncontrolled growth of abnormal cells that can invade the normal tissue boundaries, metastasize, and diffuse to other distant organs in the body^1^. According to the World Health organization, cancer is a leading cause of death worldwide. The Global Cancer Statistics reported that there were about 20 million newly diagnosed cancer cases in 2022 and nearly 9.7 million deaths globally^2,3^. Moreover, this burden is anticipated to rise to 35 million by 2035^3^. Breast cancer is one of the most prevalent malignancies and constitutes almost one-third of female cancer cases^4^. It is the leading cause of death in women all over the world^5^. Notably, breast cancer is the most frequently diagnosed women’s malignancy and the leading cause of death among women in the Middle East and North Africa (MENA) area^6^. According to GLOBOCAN 2022 database, breast cancer accounted for 25% of cancer incidence and about 20% of cancer mortality among women in MENA, with 118,200 new cases and 41,000 fatalities^6^. Although notable advancements in surgical, radio-therapeutic, and immunological methods have enhanced cancer treatment outcomes, drug treatment remains a fundamental therapeutic strategy. Nonetheless, the clinical effectiveness of drug treatment is frequently limited due to drug resistance, lack of selectivity and significant toxic side effects. Thus, the development of efficient, innovative, and selective therapies that inhibit the proliferation of cancer cells without any or lower side effects on healthy cells has become crucial to ease the burden of cancer. Indeed, cancer is still accompanied by high rates of mortality and morbidity due to its complicated, diversified nature^7^.

The ability of tumors to generate new blood vessels from already present vasculature to finally produce an integral vascular network is one of the aggressive arsenals of such tissue masses^8^ known as angiogenesis. This process is typically a complicated dynamic process that involves extended interaction between cells, extracellular matrix motifs and soluble factors^9^. It encompasses four distinguished sequential phases: basement membrane degeneration via protease enzyme, endothelial cells migration into the interstitial space and propagation, proliferation of endothelial cells and generation of novel basement membrane, lumen and anastomoses formation and eventually blood flow^9^. This vascular genesis process is controlled by diverse pro-angiogenic and anti-angiogenic factors and normally takes place in physiological conditions like wound healing, menstrual cycle and embryonic development^10^. Nevertheless, angiogenesis is well recognized as a fundamental feature of cancer progression, particularly in breast cancer^11,12^. Tumors can stimulate angiogenesis through the production of various chemical signals, including growth factors, cytokines and transcription factors^13^. Dysregulation of angiogenesis is considered a major driving factor for diverse human illnesses like cancer^14^. Tumor angiogenesis is crucial for transporting nutrients and oxygen to tumor growth and also plays an important role in delegating the metabolic dysregulation and tumor metastasis^15^. Thus, suppression of tumor angiogenesis represents as an engaging strategy for the development of promising effective cancer therapy^14^. Protein tyrosine kinases (PTKs) are enzymes that facilitate the transfer of a phosphate group from ATP to specific target proteins^13^. Protein tyrosine kinases (PTKs) serve as critical regulators of various cellular functions, including metabolism, differentiation, proliferation, transcription, and angiogenesis. These processes are also implicated in a range of diseases, including cardiovascular disease, neurodegenerative disorders, and cancer^16,17^.

Tumor angiogenesis is regulated by distinct groups of tumour-excreted growth factors (including VEGF and bFGF) through binding to their receptors on the surface of endothelial cells^9^. Vascular endothelial growth factors (VEGFs) are heparin-binding glycoproteins, expressed in several tumor types and has significant effects on the function of vascular smooth muscle cells and endothelial cells^18^. Generally, VEGFs are a glycoprotein family that includes VEGF-A, VEGF-B, VEGF-C, VEGF-D, and placental growth factor (PlGF) in mammals, amongst which VEGF-A (a diffusible glycoprotein) acts as the most biologically active one via its main binding to VEGF receptor-1 (VEGFR-1), and VEGF receptor-2 (VEGFR-2)^19,20^. Indeed, VEGFR-2 is the essential receptor that exists at the surface of the endothelial cells and is the main target for VEGF signals to mediate the angiogenesis process and endothelial cell functions^21^. Significant binding of VEGFA to the VEGFR-2 active site results in receptor dimerization followed by trans-phosphorylation of specific intracellular tyrosine amino acids, including Tyr951, Tyr1054, Tyr1059, Tyr1175, and Tyr1214. Subsequently, the phosphorylated VEGFR-2 mediates a tyrosine kinase signalling transduction cascade that eventually stimulates vasodilation, angiogenesis, cell proliferation and survival^22^.

Various disorders are closely associated with VEGFR-2, including rheumatoid arthritis and malignant neoplasms. Furthermore, VEGFR-2 is minimally expressed in healthy tissues or cells, and it has been shown that about 64.5% of metastatic breast carcinomas exhibit VEGFR-2 overexpression^23^. VEGFR-2 is aberrantly expressed, particularly in several malignant tumors, including breast cancer, ovarian cancer, and adult neuroblastoma^24^. The overexpression of VEGFR-2 may serve as a biomarker for the presence of malignant tumors. In the context of breast cancer, it was reported that VEGF-VEGFR2 signaling is crucial for the inhibition of apoptosis and the maintenance of the proliferative capability of breast cancer cells. It was reported that the elevated level of angiogenesis correlates with reduced survival in breast cancer patients^25^. Consequently, interfering with the beginning and progression of this process by targeting VEGF-VEGFR2 signaling becomes an appealing method to augment conventional therapeutic strategies for breast cancer^26^. Given these findings, the evolution of small-compound tyrosine kinase inhibitors that target the ATP binding site of VEGFR-2 to inhibit angiogenesis in tumor cells is a considerable strategy for developing safe and selective anti-cancer drugs. A diverse array of monoclonal antibodies and small-molecule inhibitors targeting VEGFR-2 have been documented as anti-cancer drugs^27,28^. Additionally, there are several small-molecule VEGFR-2 inhibitors (including Sorafenib, sunitinib, pazopanib, vandetanib, axitinib, regorafenib, cabozantinib, lenvatinib, tivozanib) that have been approved by the US FDA as anti-cancer medicines for clinical application^29^. It is noteworthy that VEGFR-2 inhibitors can be categorized into three groups: type I, type II, and type III inhibitors (30). Type I inhibitors (ATP competitive inhibitors “DFG-in conformation), including sunitinib and axitinib, could interact with the adenine region through hydrophobic interactions and involved in one to three hydrogen bonds with the adjacent amino acid residues at the VEGFR-2 active site^30^. Type II inhibitors including Sorafenib and lenvatinib, are differentiated by fitting into a hydrophobic pocket (close to ATP-binding site) and joining to the inactive "DFG-out" conformation of the kinase^31^. Type III inhibitors (covalent inhibitors), such as vatalanib, may carry out their therapeutic effects via binding covalently and irreversibly to cysteine amino acid residues at particular sites on kinases^32^. In general, the majority of these inhibitors can facilitate ongoing improvement and potentially cure certain cancer patients. Nonetheless, their clinical utilization is constrained by treatment resistance, restricted efficacy, and off-target toxicity such as hypertension and cardiotoxicity, which impede their prolonged application. Therefore, there is an immediate necessity for the evolution of novel and selective VEGFR-2 inhibitors to ride these obstacles.

Unfortunately, the conventional drug discovery and development process is a protracted endeavor, involving multiple stages to secure a unique approved medication and necessitating substantial financial commitment (about 1 billion dollars and ten years for a new drug to enter the market)^33^. Besides, drug discovery process is characterized by high rates of attrition that are primarily attributed to inadequate efficacy, insufficient toxicity profiles, or a combination of both^34^. A significant and promising method that has garnered considerable attention recently is drug repurposing as a prospective area in drug discovery process, which aims to uncover new targets and applications for clinically approved pharmaceuticals, primarily utilizing their side effects or unidentified therapeutic effects. The utilization of already approved medications shortens both the discovery and development timetable, as the conventional drug development process, encompassing pharmacology, dose formulation, and safety assessments, has previously been conducted and validated^35,36^. Conversely, medications that have received clinical approval or have been experimentally assessed for conditions unrelated to cancer, yet exhibit unforeseen lethal effects on malignant cells, may be considered promising candidates for repurposing as anti-cancer agents. Drug repurposing presents notable benefits in cancer therapy, as repurposed medications are typically economical, possess established safety records, and can expedite the therapeutic development process owing to their known safety profiles. Fortunately, numerous efficacious treatments for cancer and neurological illnesses are based on this technique^37^. For instance, thalidomide was initially prescribed as a non-barbiturate tranquilizer and has been approved for the treatment of multiple myeloma^38^. In addition, raloxifene was first introduced for the treatment of menopausal and postmenopausal osteoporosis in women and was approved for the treatment of breast cancer in 2007^39^. Moreover, meta-analysis of drugs such as metformin, statins, and aspirin revealed their correlation with a reduced cancer risk, suggesting that these treatments may be approved for cancer therapy in the near future^38^.

On the other hand, compounds that have nitrogen-containing heterocyclic moieties are amongst the most auspicious candidates for evolving novel therapeutic drugs^40^. Indeed, drugs that encompass nitrogen-containing heterocyclic scaffolds represent about 59% of all FDA-approved drugs^40,41^. They are represented as an intrinsic scaffold in the pharmaceutical industry through their diverse biological activities such as anti-fungal^42^, antibacterial^42^, anti-cancer^43^, antiviral^44^, antidepressant^45^ and anti-inflammatory^46^. Amongst the nitrogen-containing heterocyclic compounds, azoles represent a promising pharmacophore due to their structural characteristics that enable them to emulate many functional groups, validating their widespread use as bioisosters in the development of novel active compounds. Azole derivatives exhibit both hydrophobic and hydrophilic properties, possessing polar moieties at various positions. Moreover, their distinct scaffold is adaptable, allowing the introduction of various functionalities that can modulate a wide array of molecular targets involved in the proliferation of cancer disease^47^. Azole compounds, including imidazole and triazole, are reported as pharmacologically active compounds exhibiting many applications in the treatment of various diseases, including cancer. For instance, it was reported that triazole-based compound derivatives and triazole hybrids efficiently reduce VEGFR2 expression in MCF-7 breast cancer cells by obstructing angiogenesis and inhibiting tumor growth^48^ while triazole hybrids exhibit superior cytotoxicity against MCF-7 breast cancer cells compared to cisplatin, a widely utilized chemotherapeutic agent. Moreover, there are some imidazole-based pharmaceuticals, including dacarbazine, zoledronic acid, nilotinib, and tipifarnib, that have already received FDA approval as anti-cancer^49^. Tubulin, EGFR, VEGF, and tyrosine kinases are amongst the most explored targets for these azole-based anti-cancer compounds.

**Fig. 1:**
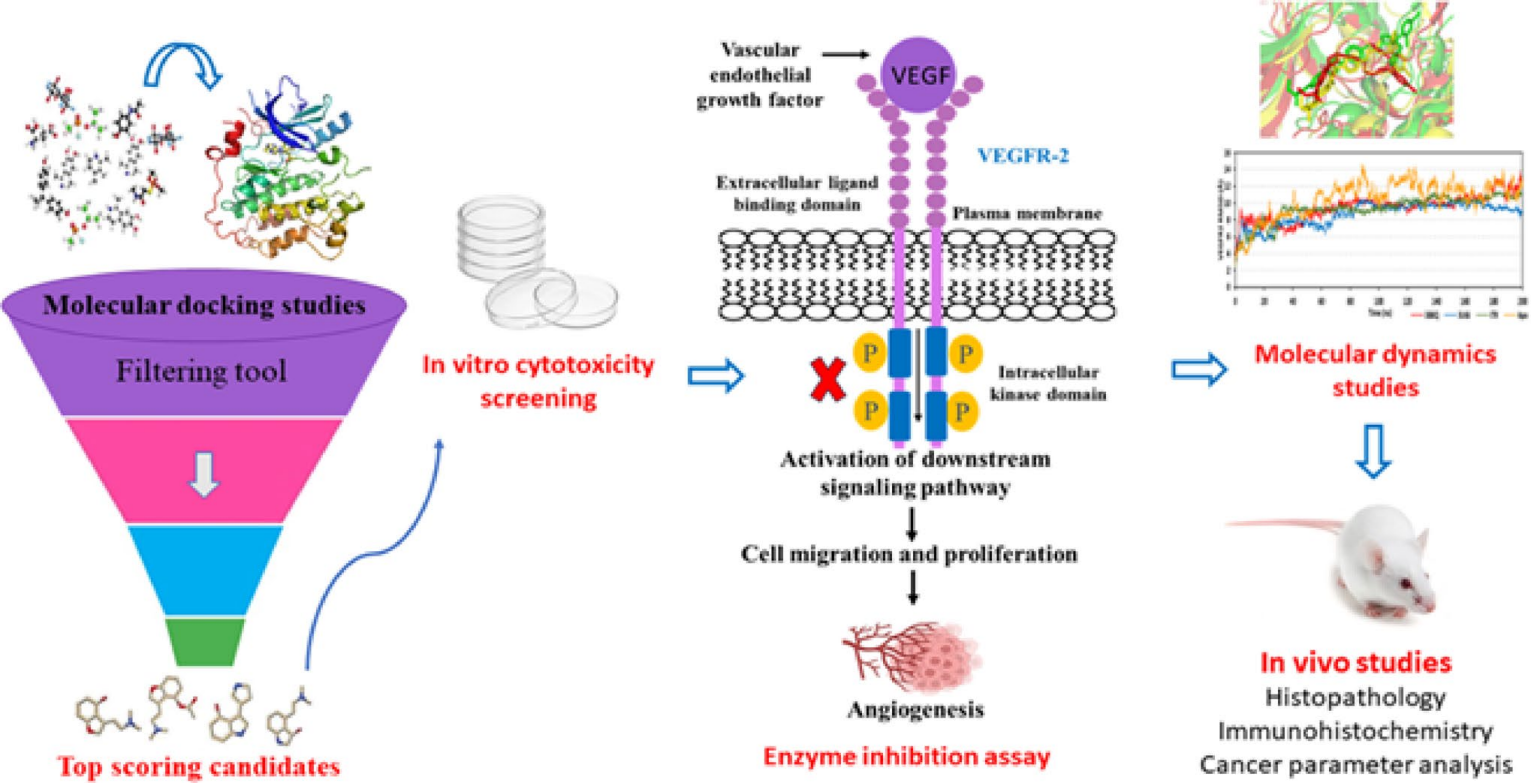
Workflow of market azoles as VEGFR-2 inhibitor and anti-cancer agent.

Given the substantial role of inhibiting VEGFR-2 in cancer treatment and the promising drug repurposing approach, our prospective study aims to conduct a comprehensive virtual screening of some market-approved azole drugs against the targeted VEGFR-2. Accordingly, molecular docking studies of the nominated compounds were performed against VEGFR-2 (Fig. 1). The top-scoring candidates were evaluated for their cytotoxic potential against the breast MCF-7 cancer cells. Furthermore, the most promising candidates were tested as potential inhibitors against VEGFR-2, followed by in vivo studies. Finally, molecular dynamics simulation studies were performed on the most promising candidate to emphasize the gained results and gain more insight about its thermodynamic characters within the protein active site that would guide future development and optimizations.

### Rationale of the work

**Fig. 2:**
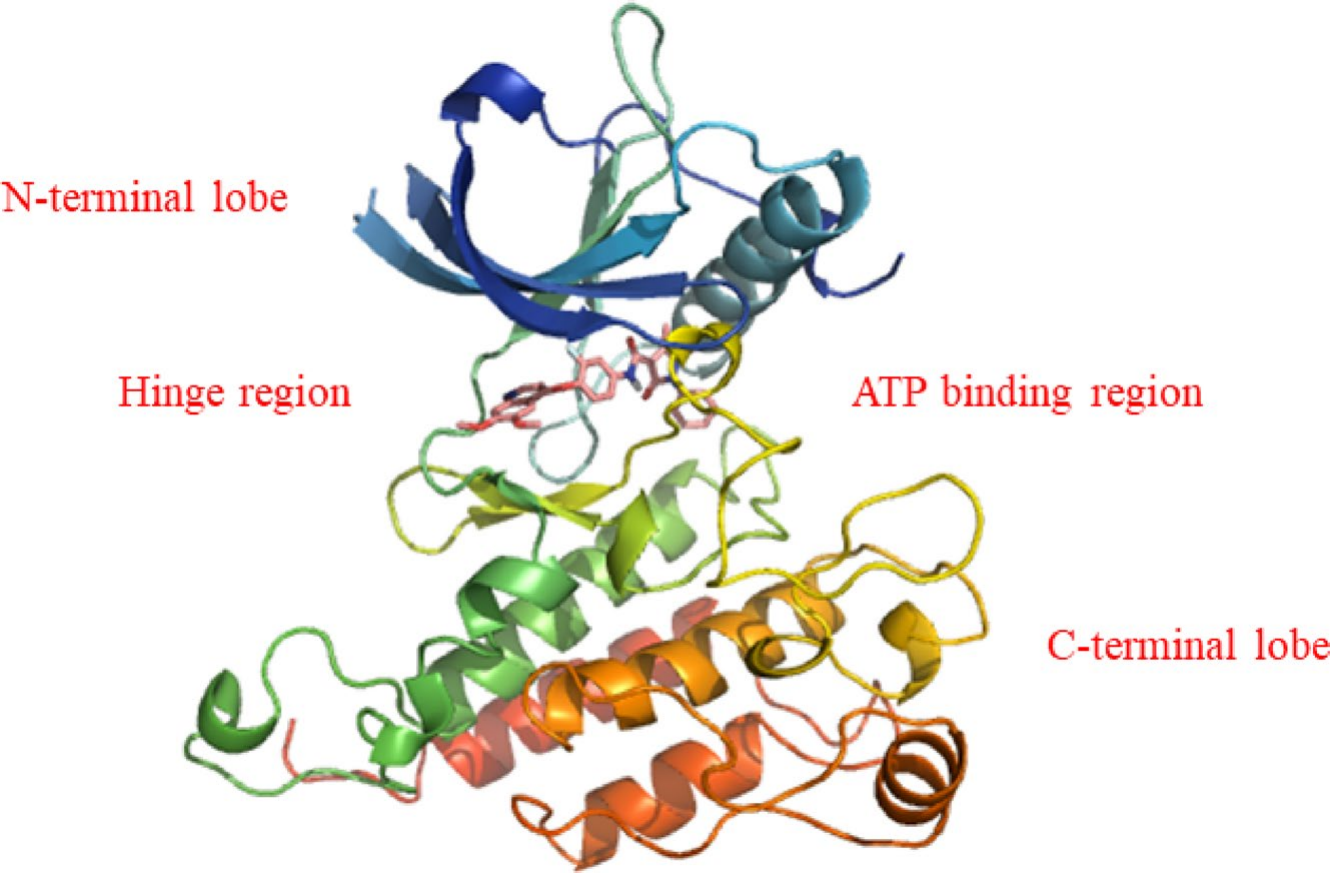
Crystal structure of VEGFR-2 (PDB: 3U6J).

The X-ray crystallographic structures of kinases, including VEGFR-2, reveal that the kinase domain consists of three compartments, including a diminutive N-terminal lobe, a more substantial C-terminal lobe, and an intervening ATP binding region, which can be further delineated into the front pocket (hinge region), gate area, and back cleft (allosteric lipophilic pocket) (Fig. 2). In addition, there is an activation loop within the beginning of the C-terminal lobe, distinguished by a conserved aspartate-phenylalanine-glycine (DFG) motif/domain. This activation loop adapts various conformations based on the 3D (three dimension) configuration of these conserved amino acid residues (DFG domain), leading to the presence of protein kinase in either its active or inactive configuration^50,51^. The preserved triad Asp-Phe-Gly (DGF) regulates the active and inactive states of kinase enzymes. Typically, the DGF-in conformation is observed in the active state, whereas the DFG-out conformation is anticipated in the inactive state^52^. The active site of VEGFR-2 consists of the front and back pockets. The ATP-binding front pocket contains two critical residues: Glu917 and Cys919. The posterior hydrophobic pocket contains Glu885 and Asp1046. Glu885 is positioned on the αC helix, while Asp1046 is a crucial component of the triad^53^.

Furthermore, the virtual screening and pharmacophore modeling investigations reported that the majority of VEGFR-2 inhibitors had four critical pharmacophoric characteristics^54^. The identified characteristics include: (1) a planar heteroaromatic ring (purine like ring) featuring a hydrogen bond acceptor that engages in hydrogen bond formation with the pivotal Glu917 and Cys919 amino acid residues in hinge region within the ATP binding domain^55^; (2) a central aromatic structure that fits into the linker region^56^; (3) a hydrogen bond acceptor and hydrogen bond donor group that occupies the DFG motif^57^; (4) an ultimate lipophilic tail situated within the back allosteric pocket^58^.

On the other hand, azoles are a class of five-membered heterocyclic compounds that include nitrogen and possess electron-rich characteristics^47^. There, distinctive structure could enable azole-based derivatives to act as a potential bioisostere of the ATP’s purine ring, enabling their binding competitively to the ATP binding pocket of VEGFR-2. Additionally, they could interact potently with the critical residues (Glu917 and/or Cys919) in the hinge region via a network of non-covalent interaction, including hydrogen bonds, electrostatic interactions, coordination bonds, and van der Waals forces.

According to the above findings, it was highly rationalized to investigate the VEGFR-2 inhibitory activity of commercially available azole drugs as potential anti-cancer agents. The latter has accomplished via assessing the efficacy of selected drugs against VEGFR-2 utilizing in silico, in vitro and in vivo studies. Implementing this drug repurposing strategy ensures medical safety, as these FDA-approved azole drugs have been assayed in animal models and have accomplished all the necessary clinical trials.

## Results and discussion

### Docking studies

Numerous VEGFR-2 kinase domains complexed with various inhibitors have been deposited in the RCSB Protein Data Bank. However, series of successive filters were carried out to pick the pertinent crystal structures for our investigation. The filters were based on the selection of crystal structures with a good X-ray Structure validation report, a good-looking Ramachandran plot, resolution less than 2.5 A° and R-Value Free not more than 0.25. Molecular docking studies of 30 market-approved azoles; etomidate (**1**), carbimazole (**2**), nilutamide (**3**), losartan (**4**), metronidazole (**5**), dantrolene (**6**), tinidazole (**7**), miconazole (**8**), secindazole (**9**), fluconazole (**10**), voriconazole (**11**), letrezole (**12**), anastrezole (**13**), itraconazole (**14**), sitagliptin (**15**), phenytoin (**16**), ethotoin (**17**), cimetidine (**18**), clonidine (**19**), oxymetazoline (**20**), naphazoline (**21**), antazoline (**22**), levamisole (**23**), vardenafil (**24**), clotrimazole (**25**), econazole (**26**), albaconazole (**27**), trapidil (**28**), deferasirex (**29**), and maraviroc (**30**) were carried out into the active site of VEGFR-2 kinase receptor (PDB: 2WZD and 3U6J).

Notably, our docking studies were performed using two protein structures with two different crystalline ligands, one exhibiting a DFG-in active conformation (2WZD) and the other a DFG-out inactive conformation (3U6J), to obtain more realistic and validated results. Re-docking of the co-crystallized ligands into its active sites was carried out to validate the used docking protocol. The produced RMSD values were 1.28, and 0.97 Å for 2WZD and 3U6J crystal structures, respectively, confirming the validation of the applied docking procedures. The docking scores of the co-crystalized ligands were -9.07 and -9.86 kcal/mole for 2WZD and 3U6J crystal structures, respectively.

Most tested azole drugs exhibited good binding affinity to the VEGFR-2 receptor and bounded via different bonding interactions. Moreover, they displayed poses that were nearly identical to those of the co-crystalised ligand. Numerous poses with good binding modes within the receptor active site were obtained, but the poses with superior RMSD refine values and docking (*S*)-score (based on its closeness to the pose of co-crystallized ligand) were nominated. The analysis of docking studies revealed that almost all tested compounds illustrated nearly comparable binding modes to the co-crystallized ligand at the active site of the targeted VEGFR-2 receptor. Additionally, most investigated compounds bind to the inactive conformation more efficiently than the active one, similar to type-II VEGFR-2 inhibitors (e.g. Sorafenib). However, itraconazole (ITR), sitagliptin (SIT), tinidazole (TNZ), fluconazole (FLU), losartan (LOS), dantrolene (DAN), and miconazole (MNZ) displayed the superior binding modes and affinity against it. Their binding scores were − 11.01, − 9.50, − 8.61, − 8.33, − 8.27, − 8.14, and − 8.03 kcal/mol, respectively. This could be attributed to the residue-wise binding interactions between these seven top-scoring azoles and the pocket residues.

Among the top-scored azoles, ITR showed a binding affinity of − 11.01 kcal/mol, better than that of the co-crystalized ligand, predicting its promising inhibitory activity against VEGFR-2 receptor. By observing the binding mode of ITR to VEGFR-2 receptor, we found that ITR is deeply fit into the VEGFR-2 receptor binding site being involved in 2 carbon hydrogen bonds with Cys919 and Thr916 through its 1,2,4-triazole-3-one, and piperazine ring. Additionally, the compound interacted with Cys1024 via a halogen bond and engaged in extensive hydrophobic interactions with Leu840, Val848, Leu1035, Ala866, Val899, Phe1047, Leu889, Ile888 and Leu1019 residues. Moreover, its dichlorophenyl moiety formed pi-cation with Arg1027 residues. Regarding SIT, its carbonyl moiety and 1,2,4 triazole ring established two significant classical hydrogen bonds with critical Cys919 and Lys868 residues. The fluorine atoms of triflorophenyl moiety and trifloromethyl engaged in three halogen bonds with Ile1044, Val899 and Cys919. In addition, there are two carbon-hydrogen bonds were formed with Asp1046 and Thr916 residues through the amino group and piperazine ring. Moreover, SIT showed significant hydrophobic interaction with diverse amino acid residues, including Leu889, Val848, Phe1047, Leu840, Ala866, and Leu1035. Concerning FLU, it demonstrated two classical hydrogen bonds with critical Cys919 and Asp1046 residues, interacted with Lys868 and Cys1045 through pi-cation interaction, and bound to Ala866, Leu1035, Leu840 and Val848 through hydrophobic interactions. Moreover, FLU is involved in a halogen bond with Glu885 and a carbon-hydrogen bond with Glu917. However, regarding the binding interactions of TNZ, it showed a significant hydrogen bond with Cys919, bound to Lys868 through a carbon-hydrogen bond and interacted with Val899, Leu1035, Leu840 and Ala866 through hydrophobic interactions as well. The detailed binding modes of the docked co-crystalized ligands and all tested azoles (**1–30**) against the two crystal VEGFR-2 protein structures are presented in Table 1S (Supplementary data). Nevertheless, the 3D-protein positioning, as well as 2D-binding interactions of the top four scored azole-based drugs (**7**, **10**, **14**, and **15**) are illustrated in Table 1.

**Table 1:**
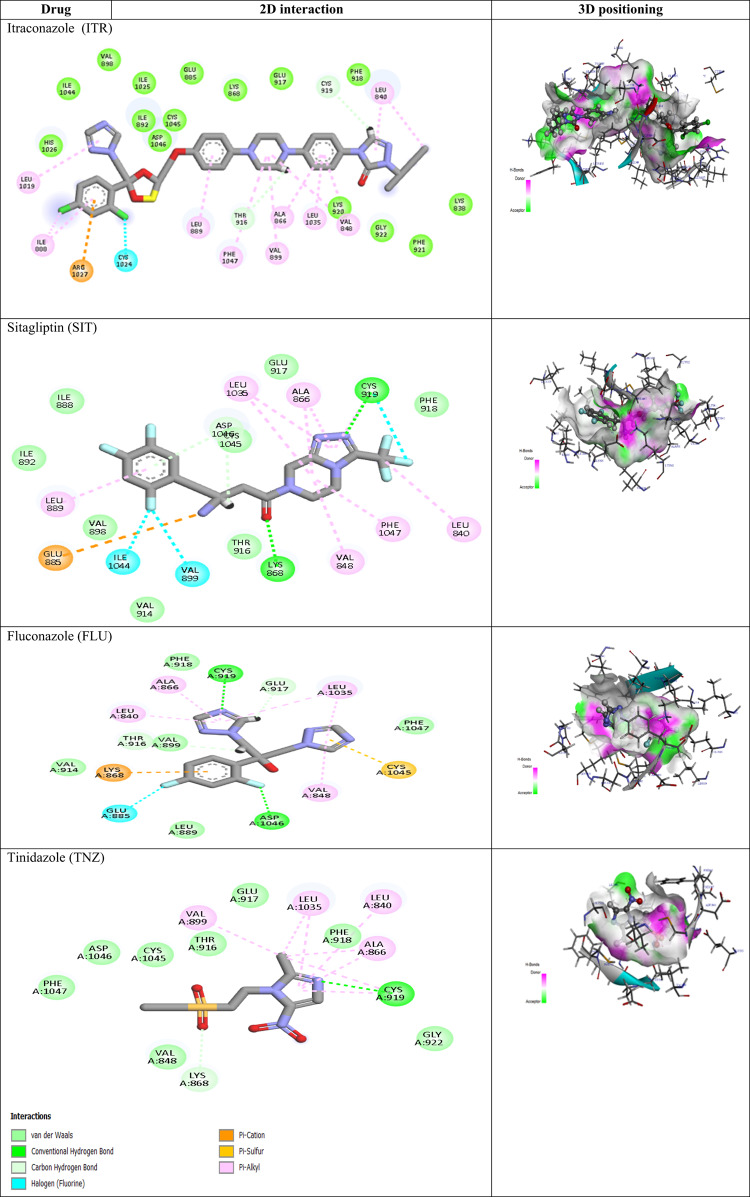
2D View of Binding Interactions and the 3D Positioning of the top four docked compounds within the VEGFR-2 Receptor Pocket (PDB: 3u6j).

### Cytotoxicity of virtually top-scored azoles against MCF-7 cancer cells in single and five dose concentrations

The seven virtual hit azoles were tested for their cytotoxicity against breast MCF-7 cancer cells and in comparison, to doxorubicin (DOX) as a positive reference control. Compounds demonstrated good cytotoxicity with a promising percentage of cell death with values of (64.6%-70.9%) at the maximum concentration [100 μg/mL], as shown in Table 2. However, based on the cytotoxicity, four azole hits were of the highest cytotoxicity profiles. To our interest, these four azoles, ITR, SIT, FLU, and TNZ, were also depicted with the top-virtual scores, which redeems their further analysis for IC_50_ cytotoxicity values. As summarized in Table 3, with the IC_50_ values of the cytotoxicity against MCF-7 cancer cells, ITR exhibited promising cytotoxicity against MCF-7 cells with an IC_50_ value of 25.74 µM compared to DOX (IC_50_ = 3.91 µM). While other compounds, SIT, FLU, and TNZ exhibited poor cytotoxicity with IC_50_ values of 183.3 µM, 260.5 µM, and 301.0 µM, respectively. Additionally, the tested compounds exhibited poor cytotoxicity against MCF-10A cells with IC_50_ range of 32.3–49.6 µM, interestingly, compound Itr showed a good safety margin against normal cells with the IC_50_ value of 49.6 µM.

**Table 2:** Cytotoxicity* of the hit azoles against MCF-7 cells at a single dose using the MTT assay.

MCF-7	Control	SIT	MNZ	DAN	FLU	LOS	ITR	TNZ	DOX
OD1	3.21	2.12	2.48	2.32	2.02	2.30	2.28	2.20	1.35
OD2	3.22	2.08	2.45	2.12	2.17	2.23	2.26	2.14	1.38
OD3	3.24	2.06	2.42	2.34	2.03	2.33	2.18	2.19	1.73
Average OD	3.22	2.08	2.45	2.26	2.07	2.29	2.24	2.16	1.49
Viability (%) 1	99.53%	65.69%	77.05%	71.99%	62.72%	71.37%	70.81%	68.18%	41.94%
Viability (%) 2	99.91%	64.42%	76.05%	65.66%	67.34%	69.14%	70.07%	66.22%	42.93%
Viability (%) 3	100.53%	63.77%	74.97%	72.49%	62.93%	72.33%	67.74%	67.96%	53.63%
Average viability%	99.99%	64.63%	76.02%	70.05%	64.33%	70.95%	69.54%	67.45%	46.16%

*Percentage of cell viability calculated from optical density (OD) in triplicates. Top-cytotoxic azoles are with yellow-highlighted average viability.

**Table 3:** Cytotoxicity in terms of IC_50_ [µm] of tested compounds against MCF-7 and MCF-10 A cells at five dose concentrations using the MTT assay.

Compound	MCF-7 IC_50_ ± SD µM*	MCF-10A IC_50_ ± SD µM*
ITR	25.74 ± 0.9	49.6 ± 2.5
SIT	183.34 ± 3.1	37.6 ± 1.9
FLU	260.55 ± 6.8	32.3 ± 1.77
TNZ	301.45 ± 7.6	51.5 ± 1.8
Dox	3.91 ± 0.6	38.9 ± 1.1

*IC_50_ values were calculated using non-linear regression curve fit using dose–response (n = 3).

### VEGFR-2 inhibition activity analysis

To gain more experimental insights into the mechanistic aspects of the four hit azoles, a Luminescent test kit was used to measure the percentage of enzyme inhibition at different doses in the study of VEGFR-2 inhibition. The rationale for investigating VEGFR-2 inhibition in MCF-7 cells as well as providing a mechanistic basis for the observed anti-breast cancer potential of itraconazole has been highlighted in current literature. Several studies have demonstrated that VEGFR-2 is not only expressed in endothelial cells but also in breast cancer cells, including the estrogen receptor–positive MCF-7 line. Both VEGFR-2 mRNA and protein have been detected in MCF-7 cells, indicating the presence of an intrinsic VEGF/VEGFR-2 signaling axis (10.1210/en.2006-0081; PMID: 15868899). Functionally, this pathway operates in autocrine and intracrine modes to promote cell proliferation, survival, and resistance to apoptosis through downstream effectors such as PI3K/AKT and p38 MAPK. Importantly, VEGFR-2 activation has been implicated in tamoxifen resistance in MCF-7-derived models, highlighting its relevance as a therapeutic target (10.1158/1541-7786.MCR-07-2172; 10.4161/cam.23332; 10.1677/erc.1.01221).

**Fig. 3:**
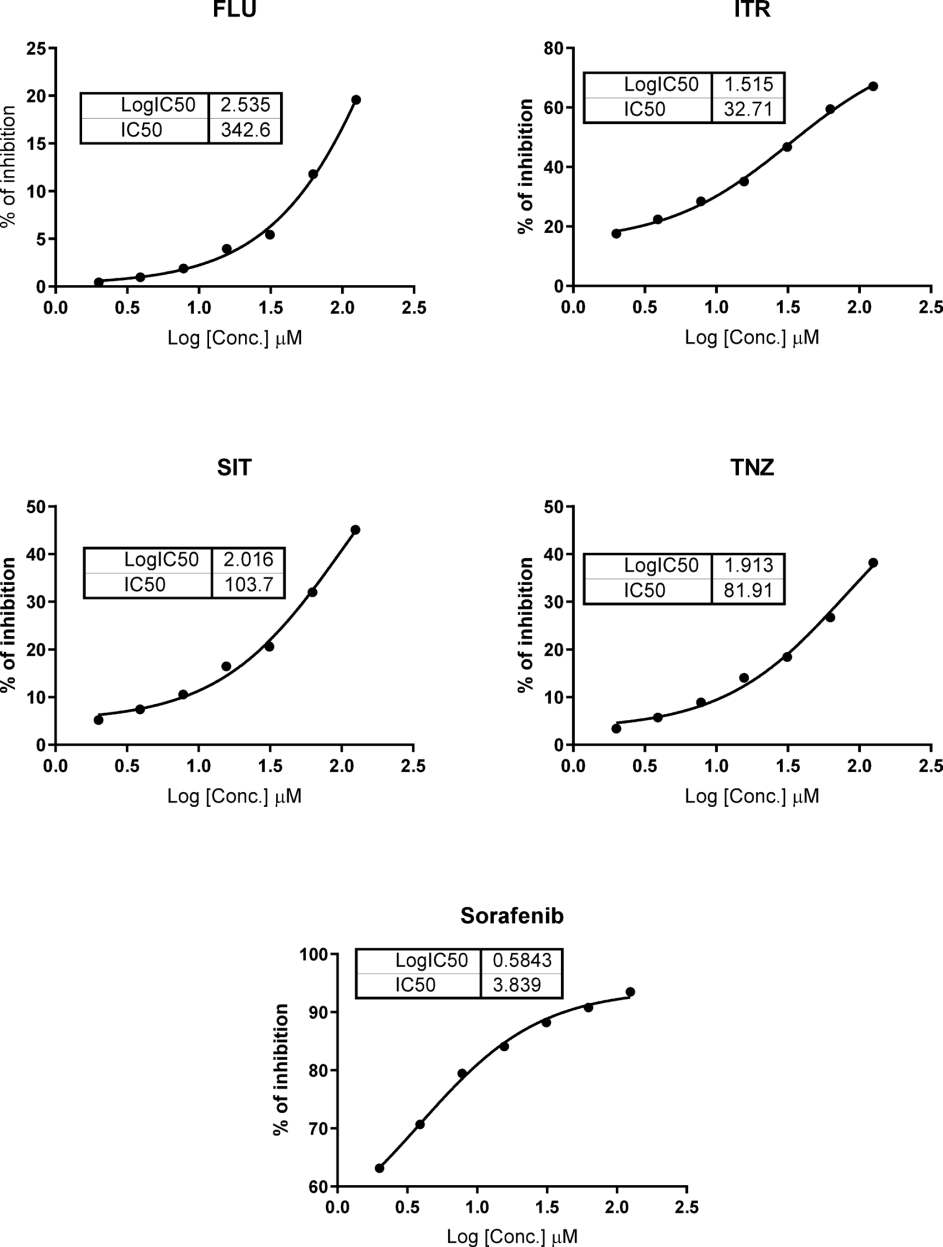
Dose–response curves of the top-cytotoxic azole compounds. VEGFR-2 Enzyme inhibition values for Itraconazole (ITR), Sitagliptin (SIT), Fluconazole (FLU), and Tinidazole (TNZ) were calculated using non-linear regression curve fit using dose–response in Graph pad prism.

As compounds Itraconazole (ITR), Sitagliptin (SIT), Fluconazole (FLU), and Tinidazole (TNZ) were the most cytotoxic agents; they were investigated for the effective molecular target. As seen in Fig. 3, interestingly, Itraconazole (ITR) exhibited promising VEGFR-2 inhibition with an IC_50_ value of 32.70 µM compared to Sorafenib (3.84 µM). Additionally, Tinidazole (TNZ) caused VEGFR-2 inhibition (IC_50_ = 81.9 nM). While Sitagliptin (SIT), Fluconazole (FLU) exhibited poor VEGFR-2 inhibition with IC_50_ values of 103.7 µM and 342.6 µM, respectively.

### Molecular dynamics studies

Based on the above iterative virtual (molecular docking) and experimental biological (cytotoxicity on MCF-7 and VEGFR-2 inhibition), the market anti-fungal azole, itraconazole (ITR), arose as a promising anti-cancer agent with promising targeting of the VEGFR-2 receptor. Thus, to gain more understanding of the ITR-VEGFR-2 activity down to the molecular level, we performed explicit molecular dynamic simulations for the anti-fungal azole at the kinase active site. Molecular dynamic simulations were performed to inspect the molecular thermodynamic behavior of ITR in relation to the bound protein, as well as compared to those of co-crystallized VEGFR2 inhibitors. This approach aimed to validate the docking findings and provide valuable molecular insights for ITR regarding its binding affinity towards VEGFR2, owing to near-real conditions offered by molecular dynamics simulations^59,60^. Conformational stability of the simulated VEGFR2 proteins, as well as those of the bounded ligands, were assessed through monitoring the root-mean square deviation (RMSD; Å) trajectories of each molecule in relation to its own reference conformation^61–63^. Typically, low-value and steady RMSD tones of the simulated protein or ligands have been correlated to stable conformation as well as relevant pocket accommodation by the ligand itself^64^.

**Fig. 4:**
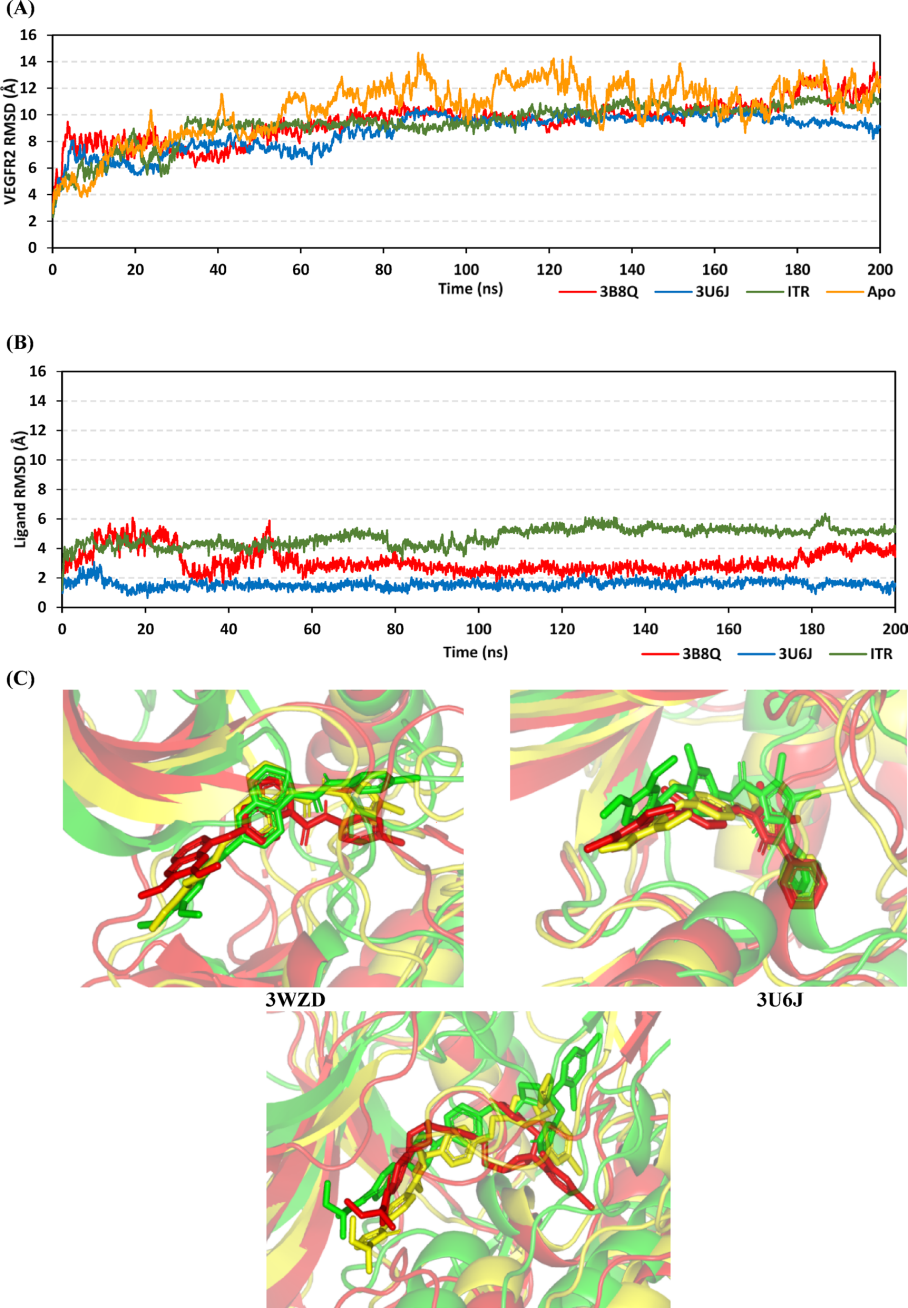

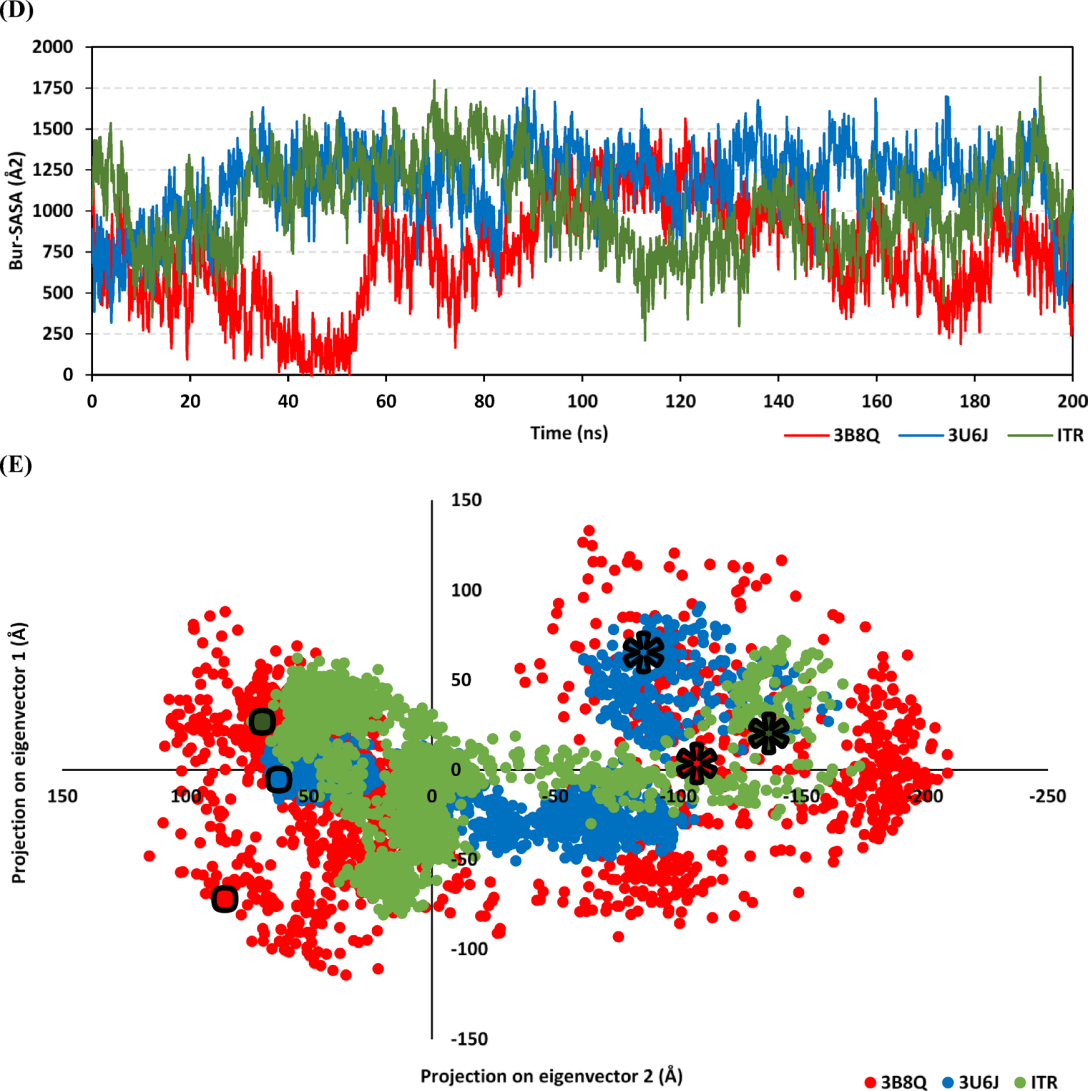
Thermodynamic and conformational stability-time evolution for the simulated compounds at VEGFR2 binding sites. (**A**) Cα-atom VEGFR2 RMSD (Å) trajectories; (**B**) Only compound RMSD (Å) tones; (**C**) Overlaid VEGFR2-compound frames at start, midway, and final simulation times. Ligands (sticks) and respective bounded VEGFR2 proteins (cartoon) are shown in green, yellow, and red colors as per initial, midway, and final extracted frames; (**D**) Bur-SASA (Å) of the simulated ligand-VEGFR2 complexes across time (ns); (**E**) PCA analysis highlighting protein atom projections within space phase along the two most dominant eigenvectors (eigenvector-1 and -2; Å^2^). Initial and final ensembles conformations are flagged by Star and Dot-labels, respectively.

Notably, RMSDs of liganded VEGFR2 were at lower values and with steadier trajectories as compared to apo (unliganded) protein state (Fig. 4A). The latter observation confers a favourable impact of ligand binding on the stability of the VEGFR2 thermodynamic behavior. Comparing the RMSD trajectories of each simulated ligand highlighted a preferential conformational stability of the 3U6J co-crystallized ligand over 3WZD and ITR since the earlier depicted lower-value and steadier RMSD tones (Fig. 4B). Great RMSD peaks and troughs were assigned for 3WZD ligand across the initial simulated times < 60 ns, before RMSD trajectories attained their own plateau till the end of the simulation run. It is worth noting that the above depicted differential ligand RMSD trajectories came in great concordance with the preceding conformational analysis across different timeframes. The top-stable co-crystallized pyrazolone inhibitor, 3U6J, showed minimal conformation/orientation alterations at the midway (100 ns) and final (200 ns) simulation frames in relation to its reference state (Fig. 4C). Interestingly, all simulated compounds depicted adequate stability at the hinge region as well as at the ATP-adenine binding pocket. Contrarily, ligand stability at the deep back hydrophobic site (selectivity pocket) was highlighted as different, with ITR showing the highest conformational/orientation alterations at the end of its simulation run.

For investigating the impact of ligand binding on VEGFR2 protein conformation evolution across time, both buried solvent-accessible surface area and principal component analysis (PCA) were evaluated. The buried solvent-accessible surface area (Bur-SASA; Å2) for each model was calculated as per the following equation (Bur-SASA = 0.5*(SASAprotein + ASAligand – SASAcomplex) to assess the time evolution conformational change at the pocket site depending on the amount of solvent being accessible at this site^65^. Additionally, Bur-SASA can provide insights regarding comparative accommodation and the extent of VEGFR2 pocket accommodation via the simulated ligands. Notably, Bur-SASA values were of great steadiness for 3U6J, yet at higher values than the other two compounds. Contrarily, 3WZD depicted the most fluctuating Bur-SASA particularly before the 60 ns timeframes, suggesting a great conformational shift across these times. Regarding ITR, fewer fluctuations for its Bur-SASA trajectories were depicted. Data from Bur-SASA analysis confers superior VEGFR2 pocket accommodation via the pyrazone inhibitor as compared to other ligands, as well as great conformational change (significant induced-fitting) for the protein ATP-binding site in bound to 3WZD (Fig. 4D). Further VEGFR2 conformational change was investigated using PCA being estimated on the protein Cα-atoms through constructing/diagonalizing the covariance matrix for capturing strenuous atomic movements via eigenvectors (overall motions of atoms) and eigenvalues (magnitude of atom’s motion contributions)^66^. Conformations of each bounded protein started from a close origin point (Star label), yet each system further evolves differently over the simulation runs till the end (Square label). As expected, the conformations of 3U6J-bounded protein converged well at the end of the simulation run, showing these conformations as close non-scattered colored dots (Fig. 4E). Similarly, the ITR-bounded system showed successful conformational convergence across the first two principal components (Projection on eigenvector 1 and Projection on eigenvector 2; Å). Contrarily, the bounded system with 3WZD showed the least convergence across the simulation times and at the end showed highly scattered dots across the two principal components. Data highlighted higher system convergence for 3U6J, followed by those of ITR and 3WZD as per reported studies^67–69^.

**Fig. 5:**
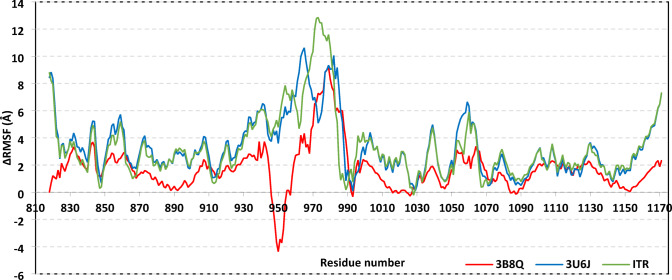
ΔRMSF analysis for bounded VEGFR2 proteins in relation to unliganded/apo one. Values are represented as per constituting amino acids (residue range; *N*-terminus Glu818 to *C*-terminus Asp1171).

Further analysis was conducted, dissecting the impact of ligand binding on protein stability down to its building units (amino acid levels). RMS-Fluctuation (Å) analysis was performed to assess the dynamic behaviours of protein residues in terms of mobility and flexibility via estimating the deviation of each residue from its reference position^70^. This analytical tool is valuable for highlighting the most stable residues and correlating them with the stability of particular ligand binding^71–73^. Notably, the difference RMSF (ΔRMSF) values were estimated as a better representation of protein mobility, where values of individual bound protein were normalized by those of the Apo protein state (PDB ID = 1VR2; atomic resolution = 2.40 Å) (i.e. ΔRMSF = RMSFUnliganded–RMSFliganded). A cut-off value of ΔRMSF = 0.30 Å has been reported relevant for estimating significant structural mobility, where amino acids assigned with values > 0.30 are considered of limited mobility^74^. At Fig. 5A, immobility patterns mainly were highlighted for the 3U6J and ITR-bounded protein as compared to those of the 3WZD system, being consistent with the above-described RMSD data. It is worth noting that both the Activation loop and Glycine-rich domain depicted the highest immobility profile with ∆RMSF up to 6.65 Å and 5.21 Å, respectively (Table 4). The latter confers the significant contribution of these residue ranges within the stability of ligands across the different domains of the binding site. An interesting finding was that the residue range (Arg946–Ala954) depicted high mobility in the case of 3WZD-bounded protein (negative-value ∆RMSF) while the same region showed high stability/rigidity patterns at the other two simulated systems. Nonetheless, this differential pattern was suggested to have less important impact on ligand binding since reported studies highlighted Thr940–Glu989 being insignificant for VEGFR2’s catalytic mechanistic activity^75^.

**Table 4:** ΔRMSF of simulated systems across the entire molecular dynamics runs.

Canonical pockets	Lining Amino acids	3WZD	3U6J	ITR
Hinge loop	Val916 (Gatekeeper)	1.02	1.75	1.40
	Glu917	1.15	2.39	2.20
	Phe918	1.57	2.82	2.52
	Cys919	1.58	3.00	2.89
	Lys920	1.92	3.71	3.50
	Phe921	1.95	3.82	3.40
	Gly922	1.68	2.97	2.37
	Asn923	1.55	2.31	1.75
	Leu924	1.51	2.52	2.01
Adenine-binding pocket	Lys838	1.90	2.99	2.08
	Thr864	2.21	2.21	1.73
	Val865	1.87	2.13	1.39
	Ala866	1.59	1.76	0.99
	Met883	0.82	2.18	2.04
Hydrophobic deep back pocket	Leu886	0.59	1.95	1.91
	Leu887	0.44	2.45	2.44
	Ile890	0.45	2.74	2.85
	Val896	0.86	2.89	3.15
	Val897	0.68	2.38	2.56
	Val912	1.63	1.08	0.75
	Leu1017	0.09	2.17	2.37
	Val1042	1.09	2.14	2.22
	Lys1043	0.76	1.58	1.65
Gly-rich loop	Gly841	2.64	3.38	2.78
	Arg842	3.50	4.72	4.29
	Gly843	3.69	5.24	4.93
	Ala844	3.33	5.21	4.63
	Phe845	1.64	3.25	2.16
	Gly846	1.20	1.96	1.05
Catalytic loop	Arg1022	0.15	2.90	3.00
	Lys1023	0.14	2.70	2.79
	Cys1024	-0.01	1.89	1.89
	Ile1025	-0.10	1.35	1.38
	His1026	-0.27	0.58	0.33
	Arg1027	-0.07	0.65	0.38
	Asp1028	0.42	0.48	-0.22
	Leu1029	0.15	0.10	0.20
	Ala1030	0.70	0.48	1.01
	Ala1031	1.07	1.34	1.59
	Arg1032	1.24	1.41	1.46
	Asn1033	0.69	0.76	0.75
Activation loop (DFG motif and nearby amino acids)	Cys1045	0.41	0.96	0.96
	Asp1046	0.25	0.84	0.77
	Phe1047	0.52	0.92	0.87
	Gly1048	0.60	1.26	1.14
	Leu1049	1.20	1.94	1.75
	Ala1050	1.67	2.37	2.05
	Arg1051	1.59	1.77	1.28

**Fig. 6:**
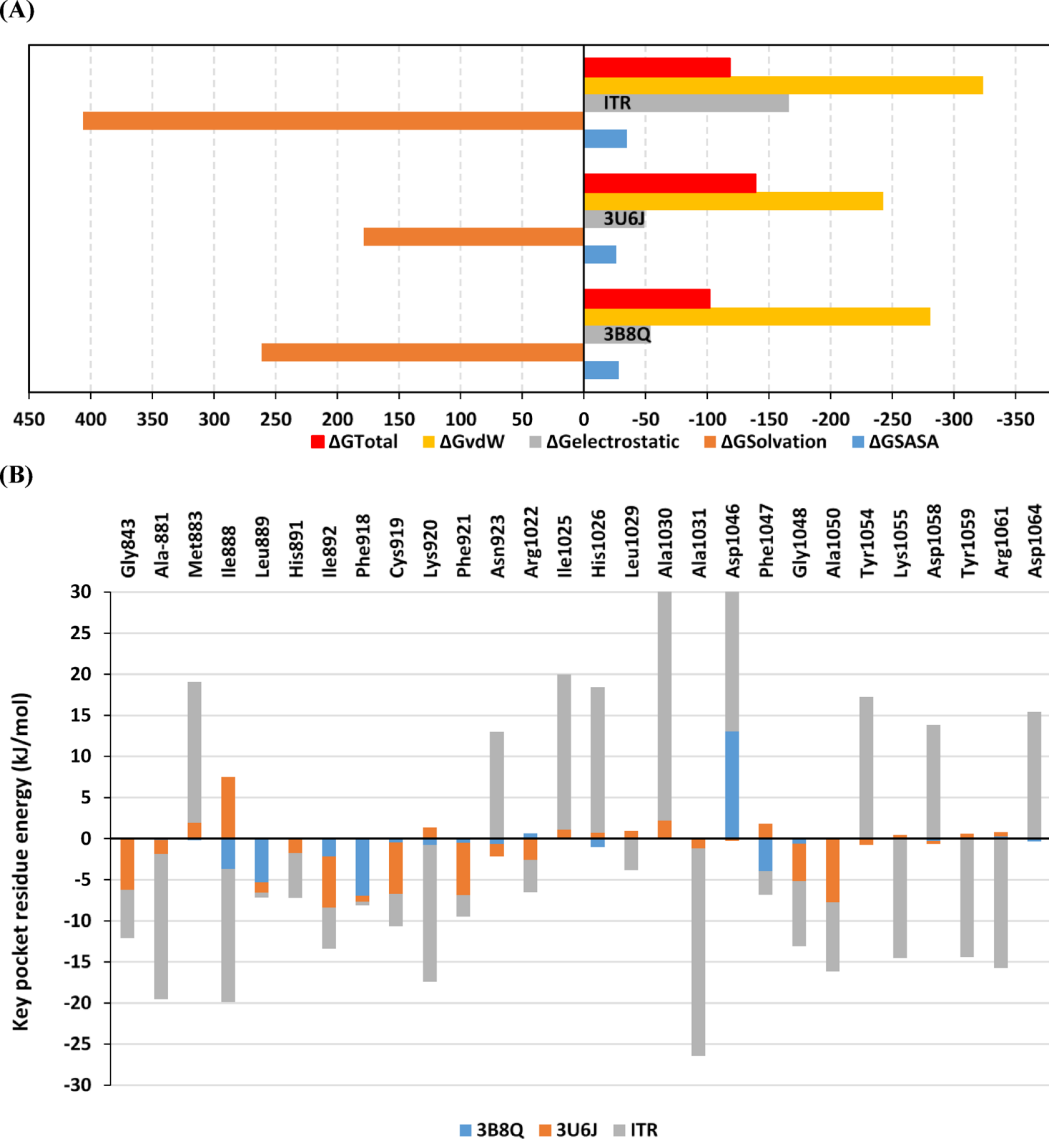
Free-binding energy for the simulated compound-VEGFR2 complexes estimated by MM_PBSA calculation. (**A**) Total free-binding energies and their dissected energy term contributions; (**B**) Residue-wise energy term contributions of the key binding residues of the important structural motifs within the VEGFR2 binding site.

Contribution of protein residues within ligand stability/binding was further depicted through estimating the Free-binding energy using the Molecular Mechanics_Poisson-Boltzmann Surface Area (MM_PBSA) calculation through the following equation (ΔGTotal = ΔGMolecular Mechanics + ΔGApolar + ΔGPolar)^76^. As a good translation of the above stability data, high-negative ΔGTotal values (higher binding affinity) were assigned for 3U6J ligand, followed by ITR and 3WZD (Fig. 6A). Dominant hydrophobic van der Waal potentials have been depicted for the energy term contribution within the total free-binding energies of the three simulated systems, with ITR being the highest. On the other hand, electrostatic potentials were almost threefold lower than the van der Waal ones at both the 3WZD and 3U6J systems. Contrarily, the van der Waal potentials at the ITR complex were just double that of the electros sic energy term. This differential energy term contribution was suggested for the advent of ITR chemical scaffolds like the triazole rings in achieving balanced hydrophobic/lipophilic characteristics^77^. Nonetheless, the ITR system was condemned with high polar solvation (ΔGPolar), which might have compromised the ITR affinity towards the VEGFR2 pocket since the binding process is a solvent replacement process^78–82^. Evaluating the residue-wise energy contributions showed significant impacts of several VEGFR2 pocket residues at different structure motifs, including Gly-loop, Cα helix, hinge domain, catalytic loop, and activation loop (Fig. 6B). It is worth mentioning that the ITR system showed higher negative-energy contributions (favoured binding) for Gly843 (Gly-loop), Ala881, Ile888 (Cα helix), Lys920 (Hinge region), Ala1031 (Catalytic loop), Lys1055, Arg1061, and Leu1067 (Activation loop). On the other hand, positive repulsive energy contributions were assigned for residues mostly present at the catalytic and activation loops. Prospective decoration of ITR skeleton with more hydrophobic functionality could be beneficial for improving hydrophobic binding potential to potentially overcompensate the depicted polar solvation entropy.

### In vivo anti-tumor activity of itraconazole in solid ehrlich carcinoma (SEC) mice

#### Tumor size and mass analysis

All experimental protocols were approved by the Research Ethics Committee and Institutional Animal Care and Use Committee, Faculty of Science, Suez Canal University with the ethical code (SCU-Sci-REC439/2025), and all methods were carried out according to the European Union guidelines and in accordance with ARRIVE guidelines. All animals were anesthetized using a combination of Xylazine 10 mg/kg- Ketamine 100 mg/kg of body weight, respectively, prior sacrifice via cervical decapitation.

**Fig. 7:**
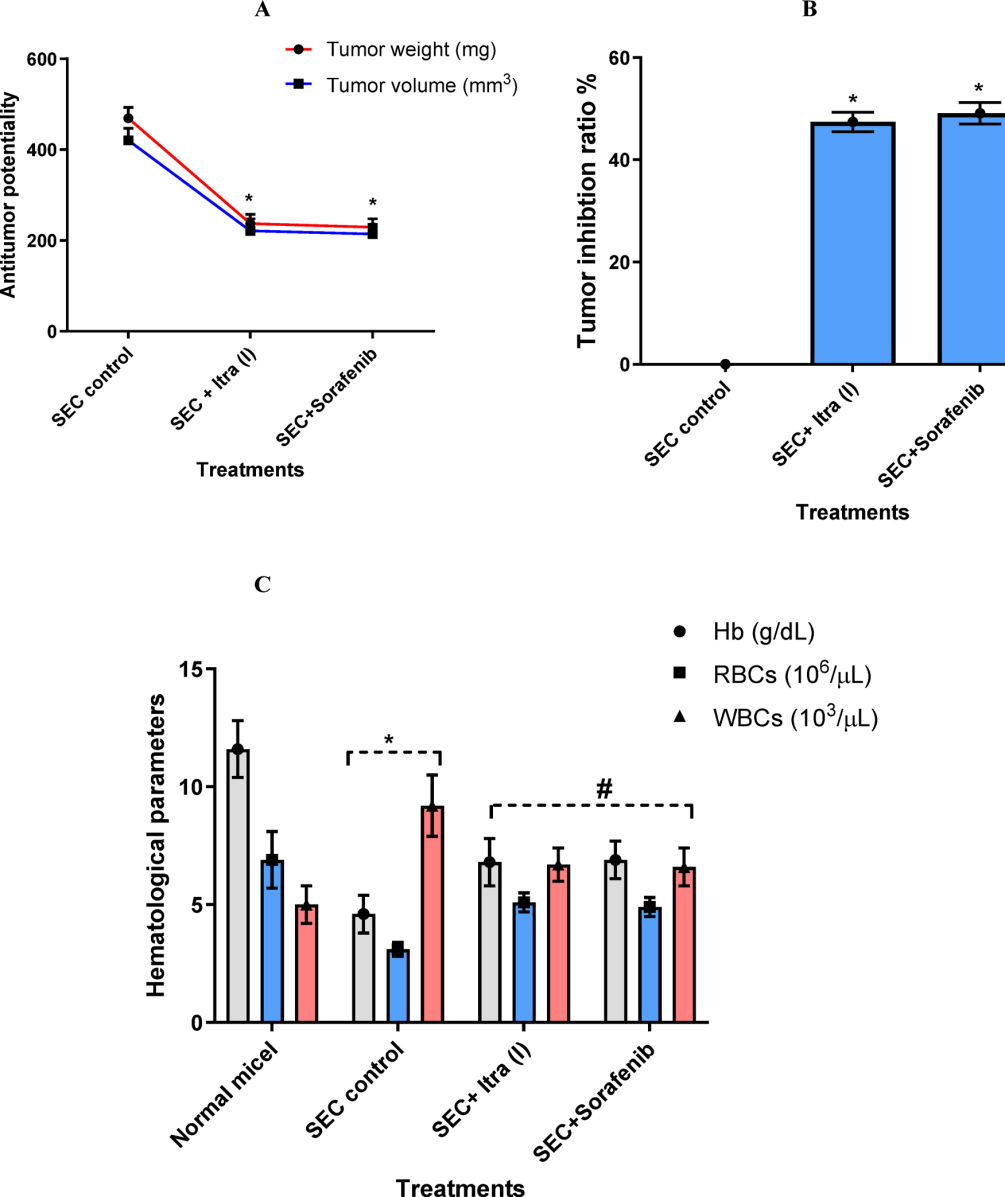
Anti-cancer activity of the compound ITR in SEC-bearing mice model in different treated groups. (**A**) Anti-tumor potentiality of compound **ITR** using tumor volume and tumor volume; (**B**) Tumor inhibition ratio (TIR%), TIR% = C − T/C × 100; (**C**) Hematological parameters in different groups of Hemoglobin (Hb, g/dL), RBC’s count (10^6^/μL), and WBC’s count (10^3^/μL). In all graphs, treatments were administrated intraperitoneal (IP) with 6 mg/Kg B.Wt. for two weeks, day after day. Values are expressed as Mean ± SD of three independent trials, *(*P* ≤ 0.05) significantly different between SEC control and normal mice, while # (*P* ≤ 0.05) significantly different between ITR-treated and SEC group using the unpaired t-test in GraphPad prism software.

Being thrilled and assured of the promising anti-cancer activity of ITR at both molecular levels, we proceeded with the biological activity analysis of this anti-fungal azole in an animal model. To our interest, ITR showed significant anti-cancer activity against the proliferation of breast cancer (Solid Ehrlich Carcinoma; SEC) in azole-treated mice. As shown in Fig. 7A, tumor mass reached an increase of 469 mg in solid mass during the experimental period. Treatment with compound ITR and Sorafenib had a significant anti-tumor effect, with solid tumor mass decreasing to 237 mg and 229 mg, respectively. Accordingly, they reduced tumor volume from 420 mm^3^ in the SEC-bearing mice to 221 mm^3^ and 214 mm^3^, respectively. Hence, both treatments inhibited tumor proliferation by 47.4% and 49.1%, respectively, which demonstrated their potency in inhibiting SEC proliferation (Fig. 7B).

Additionally, the anti-tumor potential was investigated, along with improvements in hematological parameters, through treatment. The experiment’s solid tumor propagation altered CBC parameters, including hemoglobin level, red blood cell (RBC) count, and white blood cell (WBC) count, as illustrated in Fig. 7C, when compared to normal control. Hemoglobin level in the SEC control decreased from 11.6 g/dL in normal mice to 4.6 g/dL: treatments with compound ITR and Sorafenib increased Hemoglobin levels to 6.8, 6.9 g/dL. RBC’s count was decreased from 6.9 × 10^6^/µL in normal mice to 3.1 × 10^6^/µL; treatments compound ITR and Sorafenib increased RBC’s count to 5.1 × 10^6^/µL and 4.9 × 10^6^/µL. While WBC’s count was increased from 5 × 10^3^/µL in normal mice to 9.2 × 10^3^/µL, treatments compound ITR and Sorafenib decreased WBC’s count to 6.7 × 10^3^/µL and 6.6 × 10^3^/µL, respectively. Taken together, compound ITR exhibited anti-cancer activity, improving hematological parameters in agreement with the in vitro findings regarding its selectivity profile in cytotoxicity.

#### Histopathological examination

**Fig. 8:**
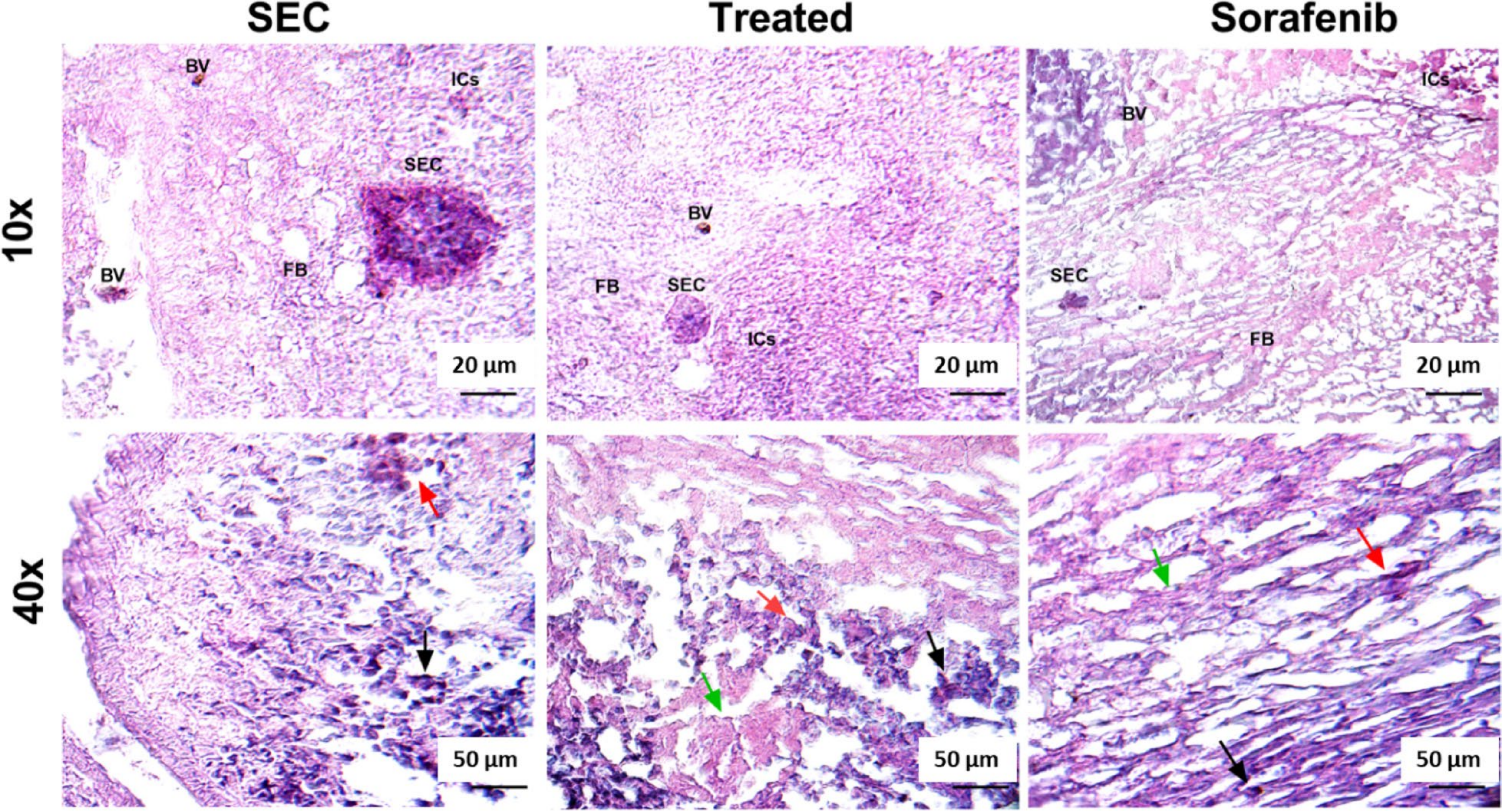
Histomorphometry of solid Ehrlich carcinoma (SEC) of mice in three different groups, SEC control, SEC treated with ITR, and SEC treated with Sorafenib as control drug. SEC: solid Ehrlich carcinoma, BV: blood vessel, FB: fibroblast cells, ICS: inflammatory cells, black arrow: pointed to hyperchromatic tumor cells, red arrow: pointed to apoptotic cells, green arrow: pointed to area of necrosis. Images are captured by the Labomed LX-400 Fluorescent (e LED) Binocular Fluorescence Compound Microscope and the Atlas 16MP Cmos USB Camera software (LABOMED, USA). The magnification power and the scale bar are presented on each image.

Histopathological examination of the isolated cancer mass from ITR-treated mice were examined in relation to those treated with Sorafenib (Fig. 8). In the control group, the solid Ehrlich carcinoma (SEC) exhibits a dense, highly cellular mass (2.2 cm^2^) with disorganized tumor architecture. The tumor cells display significant pleomorphism, characterized by hyperchromatic nuclei, an increased nuclear-to-cytoplasmic ratio, and numerous mitotic figures (indicated by the black arrow), indicating active proliferation. Angiogenesis is moderately to extensively present, with irregular and dilated blood vessels (BV) supplying the tumor. Necrotic regions are particularly noticeable in central tumor areas. The surrounding inflammatory reaction is generally mild to moderate, consisting mainly of lymphocytic infiltration (ICs) at the tumor margins. Additionally, a desmoplastic reaction is sometimes observed, characterized by fibroblast proliferation (FB) and extracellular matrix deposition (green arrow). Apoptotic features are minimal (red arrow), indicating that the tumor cells evade programmed cell death, which contributes to their continuous and aggressive growth.

Treatment with the ITR showed a notable histological change in the SEC, indicating therapeutic efficacy. Tumor cell density is visibly reduced (1.2 cm^2^) with increased evidence of apoptosis (*red arrow*), including chromatin condensation, cytoplasmic shrinkage, and apoptotic body formation. The tumor vasculature appears less developed, with a reduction in the number and size of blood vessels (BV). Necrotic regions (*green arrow*) become more extensive than in the control group. Additionally, a significant inflammatory response is observed, with increased infiltration (ICs) of macrophages and lymphocytes, suggesting an immune-mediated anti-tumor effect. The extracellular matrix undergoes fibrotic changes, leading to a denser tumor stroma characterized by the deposition of fibroblasts (FB), which may further limit tumor expansion. Mitotic figures are notably reduced (*black arrow*), indicating decreased tumor proliferation under the influence of the ITR.

Sorafenib treatment revealed a profound histological change, reflecting its strong anti-proliferative and anti-angiogenic properties. Tumor cell density is significantly decreased (0.5 cm^2^), with a marked increase in apoptotic cell death, evidenced by nuclear fragmentation and cytoplasmic condensation (*red arrow*). One of the most striking effects of Sorafenib is the disruption of tumor vasculature, resulting in severely impaired angiogenesis as detected by marked reduction in blood vessels (BV). The microvascular density is considerably reduced, and the inflammatory response is more pronounced compared to the control group, with a dense infiltration of immune cells such as macrophages and lymphocytes (ICs), which may enhance the therapeutic effect. The tumor stroma exhibits increased fibrosis (FB) and extracellular matrix deposition, contributing to a more rigid tumor microenvironment that restricts further expansion. Mitotic figures (*black arrow*) and tissue necrosis (*green arrow*) are rare, indicating that Sorafenib effectively suppresses tumor cell proliferation.

#### Immunohistochemical staining of VEGFR-2-related biomarker (VEGF)

**Fig. 9:**
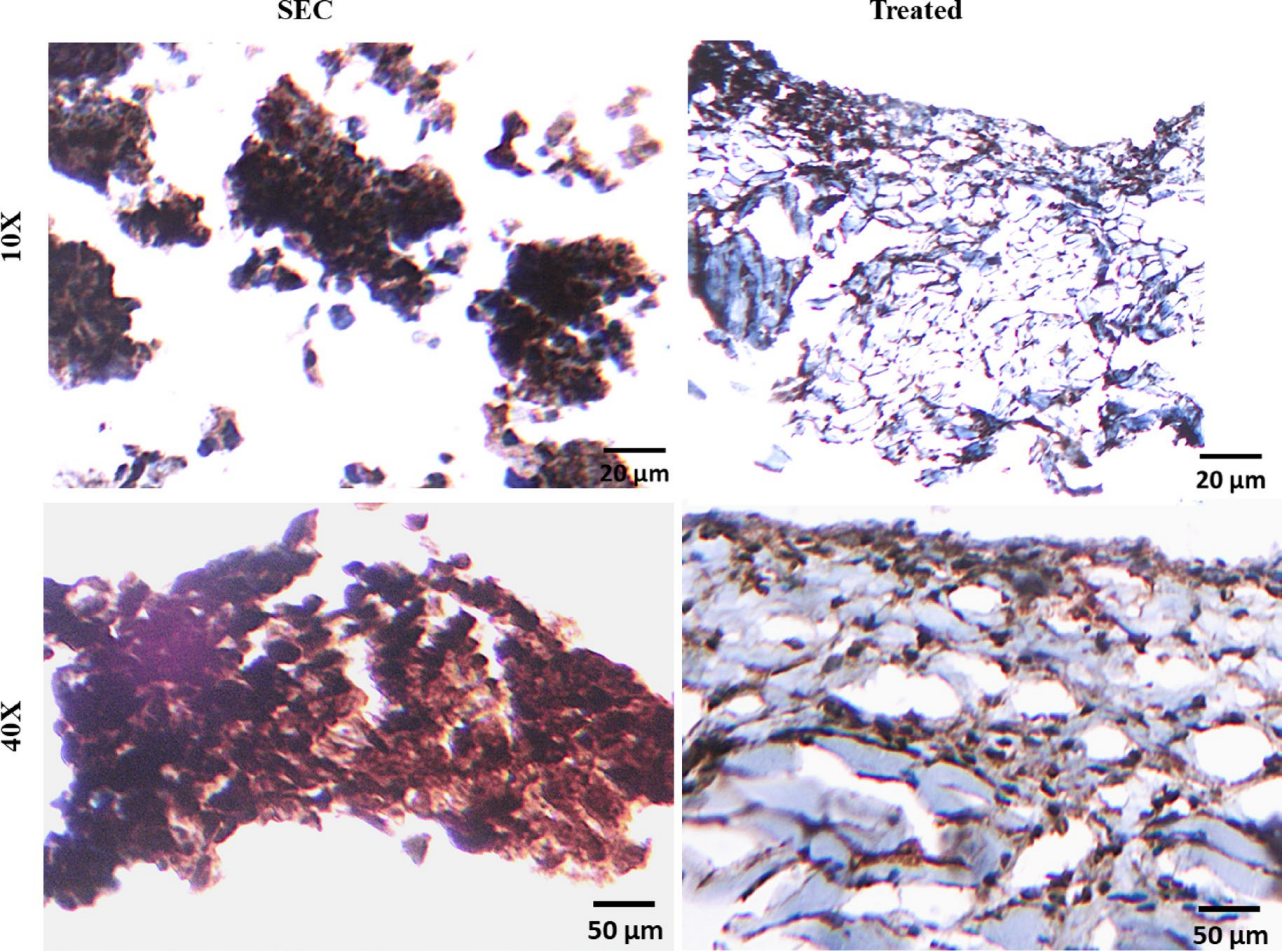
Immunohistochemical stained section of Solid Ehrlich carcinoma (SEC) tumor of mice, stained with anti-VEGF. The images show a membranous and cytoplasmic expression of VEGF in the endothelium of blood vessels and capillaries of tumor tissue. Scale bars, 20 µm and 50 µm for the magnification 10X and 40X, respectively. Images are captured by LABOMED Fluorescence microscope LX400, cat no: 9126000; US and LABOMED camera software, USA.

VEGF has been considered a biomarker for VEGFR-2 bioactivity, as it binds to and activates this kinase receptor, playing a crucial role in angiogenesis. High VEGF levels are often associated with increased VEGFR-2 expression and activity, making VEGF a marker for VEGFR-2's function and tumor growth. Immunohistochemistry (IHC) reveals that approximately 80–90% of tumor cells express VEGF (Fig. 9). The staining intensity is strong (+ 3), with dark brown cytoplasmic staining in most tumor cells. The immunoreactive score (IRS), calculated by multiplying the staining intensity score and the percentage of positive cells, is 9–12, indicating a high level of VEGF expression. This intense VEGF expression correlates with the presence of numerous irregular and dilated blood vessels in the tumor microenvironment.

Following treatment with ITR, VEGF expression is significantly reduced. The percentage of VEGF-positive tumor cells decreases to approximately 40–60%. Staining intensity is moderate (+ 2), with lighter brown cytoplasmic staining compared to the control group. The immunoreactive score (IRS) falls within the range of 4–8, reflecting a reduction in VEGF-mediated angiogenic activity. Sorafenib induced a profound suppression of VEGF expression in solid Ehrlich tumors. IHC analysis reveals that VEGF-positive tumor cells are significantly reduced to 10–30%, indicating strong anti-angiogenic effects. Staining intensity is weak (+ 1), with faint brown staining visible in only a small fraction of cells. The immunoreactive score (IRS) is significantly lowered to 1–2, indicating minimal VEGF expression.

Notably itraconazole has been reported as a possible anticancer drug, especially in relation to angiogenesis inhibition however, our study uniquely integrates comprehensive computational docking with molecular dynamics simulations, with in vitro and in vivo validation against VEGFR2. This combined strategy provides mechanistic insights into the thermodynamic stability and binding affinity of the compounds, which were not fully reported in previous studies. Previous studies generally characterized the suppression of angiogenesis by itraconazole without specifying the stability of VEGFR2 binding. Our work particularly delineates VEGFR2 as the primary target, validated by computational binding energy analysis and immunohistochemistry. The prevalence of breast cancer in the Middle East and North Africa (MENA) region is significantly elevated; nevertheless, the therapeutic potential of itraconazole has not been comprehensively investigated in this approach. Our study enhances translational value for MENA regional oncology research by concentrating on the population-centric healthcare burden and supplying region-specific data. To our best knowledge, there are few papers that have directly evaluated the efficacy of itraconazole against VEGFR2 with that of clinically approved conventional anti-angiogenic agents. Our comparative results with the conventional breast cancer therapy drug, sorafenib, indicate that itraconazole exhibits considerable tumor suppression indices, presenting it as a promising candidate for repurposing with potential safety and cost benefits. Ultimately, our work underscores the drug-repurposing strategy, underlining itraconazole’s known pharmacokinetics and safety profile. This study enhances the narrative on itraconazole’s anticancer effects by situating it within a comprehensive context of cost-effective, fast translatable therapeutic alternatives, which is increasingly pertinent to global health systems.

Given these considerations, we assert that the manuscript presents novel discoveries by elucidating the mechanism of itraconazole’s inhibition of VEGFR-2, demonstrating its efficacy in both computational and experimental models, and providing a comparison analysis with sorafenib.

## Material and methods

The molecular docking workflow were carried out using Auto Dock Vina V.1.2.0 (Scripps Research, La Jolla, CA, United States). Pose visualization and compounds binding interactions were conducted using PyMol V2.0.6 (Schrödinger, NY, USA). The target proteins (**3wzd** and **3u6j**) were obtained from the RCSB Protein Data Bank. Molecular dynamics simulations for itraconazole (ITR) as compared to co-crystallized VEGFR2 inhibitor in complex to VEGFR2 were performed using GROMACS-2019 software package under CHARMM36m and CHARMM-General forcefields as per reported study. All tested compounds of SIT, MNZ, DAN, FLU, LOS, ITR and TNZ were tested preliminary for their cytotoxicity against the MCF-7 cells using the single dose, then the promising compounds ITR

SIT, FLU and TNZ were tested using the five dose sigmoidal curves to determine the IC_50_ values. They were tested for the VEGFR2 inhibition using VEGFR2 Kinase Inhibition Assay Kit Catalog # 40325 BPS Bioscience, San Diego, CA. Finally, the lead compound (ITR) was further tested using the SEC-bearing mice dividing the groups into three; SEC control, SEC+ITR and SEC+ Sorafenib. At the end of these experiment, anti-tumor potentiality was recorded using tumor weight (mg), tumor volume (mm^3^), and the tumor inhibition ratio (TIR%). Hematological parameters of Hemoglobin (Hb, g/dL), RBC’s count (10^6^/μL), and WBC’s count (10^3^/μL) were investigated, along with histopathological and immunohistochemical staining of the VEGR2 protein.

## Conclusion

Repurposing existing azole compounds through advanced computational and experimental approaches offers a promising strategy for cancer therapy. Molecular modeling reveals that these azoles engage with key cancer-related targets through high-affinity interactions. At the same time, biological assays demonstrate selective cytotoxicity against cancer cells, particularly ITR, which exhibits promising cytotoxicity against MCF-7 breast cancer, with an IC_50_ value of 25.74 µM. Additionally, it inhibits the VEGFR2 target enzyme with an IC_50_ value of 32.71 µM, compared to Sorafenib. In an in vivo model using the SEC-bearing model, ITR treatment exhibited potent anti-cancer activity, resulting in a significant reduction of the tumor inhibition ratio by 47.4% compared to Sorafenib, at 49.1%. It ameliorated hematological parameters in agreement with the in vitro findings regarding its selectivity profile in cytotoxicity. This approach leverages existing pharmacological data on approved azoles to accelerate the development of novel anti-cancer therapies, thereby reducing discovery timelines.

## Supplementary Information

Below is the link to the electronic supplementary material.

Supplementary Material 1

## Data Availability

All data associated with the manuscript are available upon reasonable request from the corresponding author (Asmaa M. Atta, [asmaa-mohamed@buc.edu.eg](mailto:asmaa-mohamed@buc.edu.eg)).
