# Supplementary material for: Exploring market-approved azoles as potential breast cancer therapeutics targeting the VEGFR-2 biotarget
Source: Sci Rep. 2025 Dec 10;15:43581. doi: 10.1038/s41598-025-29322-6 (PMC12699035; doi:10.1038/s41598-025-29322-6)
Supplement: Supplementary file 1 — Supplementary Material 1 [file 41598_2025_29322_MOESM1_ESM.docx]

**Supplementary data**

**Table (1S).** The detailed binding modes of the docked co-crystalized ligands and all tested azole drugs (1-30) against the two crystal VEGFR-2 protein structures

| **No.** | **Drug name** | **(3WZD)**  **S-**  **Score**  **DFG-in** | **Interactions with**  **binding site** | **(3u6j)**  **S-**  **Score**  **DFG-out** | **Interactions with**  **binding site** |
| --- | --- | --- | --- | --- | --- |
| 1 | Etomidate | -5.99 | CYS1045 H-acceptor  ASP1046 H-acceptor  VAL 848 pi-H | -6.72 | CYS1045 H-acceptor  ASP 1046 H-acceptor  CYS1045 pi-H |
| 2 | Carbimazole | -4.52 | LEU 840 pi-H | -5.59 | CYS1045 H-acceptor  ASP 1046 H-acceptor |
| 3 | Nilutamide | -6.11 | LEU 840 pi-H | -6.44 | THR 916 H-donor  LYS 868 H-acceptor |
| 4 | Losartan | -7.43 | VAL914 H-donor  PHE918 H-acceptor  LEU 840 pi-H  LEU 1035 pi-H | -8.27 | LYS 868 Ionic  PHE 1047 H-pi  LYS 868 pi-H  LYS 868 pi-cation  ASP 1046 pi-H  PHE 1047 pi-H |
| 5 | Metronidazole | -4.62 | ASP1046 H-acceptor | -5.30 | LYS 868 H-acceptor  ASP 1046 pi-H |
| 6 | Dantrolene | -7.77 | ASP1046 H-donor  ASP1046 H-acceptor | -8.14 | CYS 1024 H-donor  ASP 1046 H-acceptor  CYS 1045 pi-H |
| 7 | Tinidazole | -7.65 | CYS919 H-acceptor  LEU840 pi-H | -8.61 | CYS919 H-acceptor  LYS868 H-acceptor  VAL899 pi-H  LEU1035 pi-H  LEU840 pi-H  ALA866 pi-H |
| 8 | Miconazole | -7.21 | CYS1045 H-donor  LEU 840 pi-H  LEU 840 pi-H | -8.03 | ASP 1046 H-acceptor  LYS 868 pi-cation |
| 9 | Secindazole | -5.56 | GLU885 H-donor  ASP1046 H-donor | -5.95 | ASP 1046 H-acceptor  LYS 868 pi-cation |
| 10 | Fluconazole | -6.98 | CYS1045 H-donor  LYS868 pi-H | -8.33 | CYS919 H- acceptor  ASP1046 H- acceptor  GLU917 H- donor  LYS868 pi-cation  CYS1045 pi-cation  ALA866 pi-H  LEU1035 pi-H  LEU840 pi-H  VAL848 pi-H  GLU885 Halogen  bond |
| 11 | Voriconazole | -5.75 | GLY 846 H-acceptor  GLY 922 pi-H  LEU1035 pi-H | -6.18 | ASP 1046 H-donor  LYS 868 H-acceptor  LEU 889 pi-H  PHE 1047 pi-pi |
| 12 | Letrezole | -5.38 | LEU840 pi-H | -5.69 | CYS 1045 pi-H  ASP 1046 pi-H |
| 13 | Anastrezole | -5.86 | ASP1046 H-donor | -6.36 | CYS 1045 pi-H  CYS 1045 pi-H |
| 14 | Itraconazole | -10.14 | CYS1045 H-acceptor  ASP1046 H-acceptor  LEU840 pi-H | -11.01 | CYS 919 H-donor  THR 916 H-donor  LEU 840 pi-H  ILE 888 pi-H  VAL848 pi-H  LEU1035 pi-H  ALA866 pi-H  VAL899 pi-H  PHE1047 pi-H  LEU889 pi-H  LEU1019 pi-H  ARG1027 pi-cation  CYS1024 halogen  bond |
| 15 | Sitagliptin | -8.85 | CYS1045 H-donor  ASP1046 H-donor  CYS919 H-acceptor  LEU840 pi-H | -9.50 | CYS 919 H-acceptor  LYS868 H-acceptor  ASP1046 H-donor  THR916 H-donor  ILE1044 halogen  bond  VAL899 halogen  bond  CYS919 halogen  Bond  LEU889  VAL848 pi-H  PHE1047 pi-H  LEU840 pi-H  ALA866 pi-H  LEU1035 pi-H |
| 16 | Phenytoin | -5.13 | CYS1045 H-donor  CYS1045 H-donor  VAL848 pi-H | -5.97 | CYS 1045 pi-H |
| 17 | Ethotoin | -4.85 | LEU840 pi-H  LEU840 pi-H | -6.19 | ASP 1046 H-acceptor  LYS 868 pi-H |
| 18 | Cimetidine | -6.20 | CYS919 H-donor  ASP1046 H-donor | -6.98 | CYS1045 H-acceptor  ASP1046 H-acceptor  ASP1046 pi-H |
| 19 | Clonidine | -5.15 | GLU917 H-donor  LEU840 pi-H | -6.03 | ASP 1046 H-donor  THR 916 H-donor |
| 20 | Oxymetazoline | -6.09 | ASP1046 H-donor  VAL848 pi-H | -6.90 | ASP 1046 H-donor  THR 916 H-donor  ASP 1046 pi-H |
| 21 | Naphazoline | -5.37 | CYS1045 H-donor  LEU 840 pi-H | -6.08 | LYS 868 pi-H  LYS 868 pi-H  LYS 868 pi-cation |
| 22 | Antazoline | -5.82 | CYS1045 pi-H  ASP1046 pi-H | -6.55 | LYS 868 pi-H  LYS 868 pi-cation  CYS 1045 pi-cation |
| 23 | Levamisole | -5.26 | GLU885 H-donor  VAL848 pi-H | -5.71 | LYS 868 pi-H  LYS 868 pi-cation  PHE 1047 pi-H |
| 24 | Vardenafil | -7.58 | ASP1046 H-donor  GLU885 H-donor  LEU840 pi-H | -10.04 | LYS 868 pi-cation  ASP 1046 pi-H |
| 25 | Clotrimazole | -5.40 | GLY846 H-acceptor  LYS868 H-acceptor  GLY841 pi-H | -2.33 | CYS 919 H-acceptor |
| 26 | Econazole | -6.60 | GLU917 H-donor  LYS868 pi-H | -7.75 | ASP 1046 H-donor  CYS 1045 pi-H |
| 27 | Albaconazole | -6.61 | ASP1046 H-donor  LEU840 pi-H  CYS1045 pi-H | -8.51 | ASP 1046 H-acceptor  ILE 892 pi-H |
| 28 | Trapidil | -5.61 | CYS919 H-acceptor  LEU840 pi-H  LEU840 pi-H | -5.99 | LEU 889 pi-H  ASP 1046 pi-H |
| 29 | Deferasirex | -7.23 | ASP1046 H-donor  VAL848 pi-H  VAL848 pi-H | -8.02 | CYS 1024 H-donor  ARG 1027 Ionic  ASP 1046 pi-H |
| 30 | Maraviroc | -7.43 | ASP1046 H-donor  PHE918 H-acceptor  LEU840 pi-H | -8.64 | ASP 1046 H-donor  ASP 1046 H-donor  LEU 889 pi-H |
| 31 | Co-crystalized ligand | -9.07 | GLU 885 H-donor  ASP 1046 H-acceptor  LEU 840 pi-H  LYS 868 pi-H | -9.86 | GLU 917 H-donor  ASP 1046 H-donor  CYS 919 H-acceptor  PHE 1047 H-pi |

**Materials and methods**

**Molecular modelling studies**

The molecular docking workflow were carried out using Auto Dock Vina V.1.2.0 (Scripps Research, La Jolla, CA, United States). The binding site was characterized by the co-crystallized ligand, and the docking was carried out using the Vina Forcefield and Lamarckian Genetics, with the biological target center serving as the docking box center. Global search exhaustiveness was established at 100 kcal/mol, whereas the maximal energy variation between poses were fixed at 3 kcal/mol. Pose visualization and compound/Mpro binding interactions were conducted using PyMol V2.0.6 (Schrödinger, NY, USA). The target proteins (**3wzd** and **3u6j**) were obtained from the RCSB Protein Data Bank. The Auto Dock Tool package v1.2.0 was utilized to structurally prepare the downloaded protein by eliminating co-crystallized ligand, solvent, and ions, incorporating polar hydrogen atoms and partial charges, and merging non-polar hydrogens absent from the X-ray crystallized PDB file. The generated target proteins were subsequently transformed into PDBQT file format for subsequent utilization. Synthesized compounds were generated, transformed into 3D structures, energy minimized using AMBER partial charges/modified forcefield, and subsequently converted into PDBQT files utilizing Open Babel tool v.2.3.1 (National Supercomputer Centre, Linköping, Sweden).

Molecular dynamics simulations for itraconazole (ITR) as compared to co-crystallized VEGFR2 inhibitors (PDB ID: 2WZD and 3U6J) in complex to VEGFR2 were performed using GROMACS-2019 software package under CHARMM36m and CHARMM-General forcefields as per reported study [1]. In brief, each complex was individually solvated in TIP3P cube box at conditions of periodic boundaries with marginal distances of 10Å [2]. Net charge of the whole system was adjusted via adequate number of K+ and Cl- atoms [3]. Each system was minimized at 5ps steepest-descent algorithm [4], followed by double equilibration ensembles (100ps each) under NVT (303.15K) then NPT (1 atm. P; 303.15K) ensembles [5]. Equilibrated systems were then run for 200ns under explicit conditions of NPT ensemble. Particle Mesh Ewald algorithm was adopted to compute the far-range electrostatic contacts [6], while as, covalent bonds were modeled using LINCS at 2fs integrated timestep sizes [7]. Verlet cut-off at 10Å was adopted for van der Waals and Coulomb’s interactions [8]. Ligand-protein binding-free energy was estimated via MM_PBSA calculations [9].

**Biological Assay**

**Cancer Cell Lines and Culture Conditions**

Breast cancer MCF-7 cells were purchased from the American Type Culture Collection ATCC. Cells were seeded at a density of 8000 cells per well in high glucose Dulbecco’s modified eagle medium (DMEM) (Invitrogen, USA), as previously described [10]. The 3-(4,5-dimethylthiazol-2yl)-2,5-diphenyl tetrazolium bromide (MTT) colorimetric assay was used to assess the antiproliferation activity of the examined compounds, as previously published [11].

**VEGFR-2 enzyme activity**

Compounds Itraconazole. Sitagliptin, Fluconazole and Tinidazol**e** were tested for their ability to inhibit VEGFR-2 kinase activity using a VEGFR2 (KDR) kinase assay kit (BPS Bioscience, Corporation catalog # 40325) according to the manufacturer's instructions. The percentage inhibition of autophosphorylation by substances was estimated using the following equation: Percentage inhibition = $100-[\frac{Control}{Treated}-Control)]$ [12].

***In Vivo* anticancer assay in SEC mice**

***Animals and tumor cell lines***

Adult female Swiss albino mice purchased from Faculty of Pharmacy, Suez Canal University, Ismailia, Egypt, with an average body weight of (18-23) g was used. Mice were housed under constant conditions of 12 h light/dark cycle in a temperature under conditions of controlled humidity (22 ± 2 °C), with free access to standard laboratory mice food and water. All procedures related to care and maintenance of the animals were performed according to the international guiding principles for animal research and approved by the Faculty of Pharmacy, Suez Canal University bioethics and animal ethics committee.

Solid Ehrlich carcinoma (SEC) was got from the National Cancer Institute (Cairo University, Egypt). The tumor cell line proliferated in mice through serial intraperitoneal (I.P.) transplantation of a volume of 0.2 mL physiological saline containing 1×10^6^ viable cells for 24 h. SEC cells were collected 7 days after I.P. implantation. The harvested cells were diluted with saline to obtain a concentration of 5×10^6^ viable SEC cells/mL. A volume of 0.2 mL saline contains 1×10^6^ SEC cells that were I.P. implanted into each normal mouse. SEC cells (1×10^6^ tumor cells/mouse) were implanted subcutaneously into the right thigh of the hind limb.

The experimental animals were randomly divided into four groups. Group **1** served as the normal saline control. Group **2** served as the SEC control (1×10^6^ cells/mouse). Group **3** served as the ITR-treated group (6 mg/kg B.Wt., I.P.). Group **4** received the standard anticancer drug of Sorafenib (6 mg/kg BW, I.P.) and is considered as a reference control. Body weight and survival were recorded daily until the 24^th^ day in both treated and control groups. At the end of the experiment, anesthetized animals were then sacrificed for evaluation of the antitumor activity and histopathological examination.

***Antitumor potentiality***

It includes tumor volume, weight, and tumor inhibition ratio (TIR%). Time interval measurements of tumor volume using a digital Vernier caliper (Tricle Brand, Shanghai, China). Measure tumor length and width using a clipper and then calculate tumor volume using formulations V = (L x W x W)/2, where V is tumor volume, W is tumor width, L is tumor length. While TIR% was calculated according to the following equation $\frac{Tumor volume \left( Control \right)-Tumor volume (treated)}{Tumor volume (control)}x 100$.

***Histopathological study***

Specimens of liver-sacrificed mice were fixed in 10% saline formalin. The fixed liver specimens were Dehydrated in ascending series of ethyl alcohol and embedded in paraffin. Sections at 5 mm thickness were stained with hematoxylin and eosin and examined under light microscopy.

***Immunohistochemistry staining Protocol for FFPT Sections***

The paraffin-infiltrated tissue was sliced into thin sections (4-6 μm) using a microtome and floated in a water bath. The sections were then mounted onto charged slides and left to dry overnight. The use of charged slides ensures the sections adhere properly to the slide. To carry out antibody staining, the paraffin wax was removed from the sample, and the tissue was rehydrated. The paraffin was eliminated by placing the tissue sections in three containers of xylene for 5 minutes each. Rehydration began by immersing the sections in two containers of 100% ethanol for 10 minutes each, followed by two containers of 95% ethanol for 10 minutes each. Finally, to complete the rehydration, the sections were washed twice in deionized water for 5 minutes each.

For antigen retrieval, the slides are heated in 10 mM Tris/1 mM EDTA, pH 9.0, kept at a sub-boiling temperature for 18 minutes, and then allowed to cool at room temperature for 30 minutes. For chromogenic staining detection, the sections are rinsed in deionized water three times for 5 minutes each, and then the endogenous peroxidase activity in the tissue sections is quenched to prevent high background staining by incubating the sections in 3% hydrogen peroxide for 10 minutes. Afterward, the sections are washed twice in deionized water for 5 minutes each, followed by a single wash in wash buffer for 5 minutes. A hydrophobic pen is used to carefully draw a large circle around the sample, ensuring it doesn’t touch the sample, creating a hydrophobic boundary to achieve optimal antibody staining quality. To prevent non-specific antibodies from binding to the tissue, each section is blocked with 100–400 µl of blocking solution for 1 hour at room temperature in a humidified chamber. The blocking solution was discarded, and 100–400 µl of diluted primary antibody was applied. The sections were then stained with protein antibodies for *BCL-2 Recombinant Rabbit Monoclonal Antibody (ARC0173), (Product #* *MA5-35345),* *TSC22D1 Polyclonal Antibody (Product #* *TL26991763),* and *EGFR Mouse Monoclonal Antibody [Product # B6-E5-D9] (Invitrogen, Thermosientific, USA)*, at dilution of 1:250.

The slides were incubated overnight at 4°C in a humidified chamber. The following day, the immunohistochemical detection kits were brought to room temperature. The primary antibody solution was discarded, and the sections were washed three times with wash buffer, each wash lasting 5 minutes. Then, 1–3 drops were added to cover the tissue sections. *EnVision FLEX link Detection Reagent, cat no: K8000 (Dako, Denmark)*. The slides were incubated in a humidified chamber for 30 minutes at room temperature, then washed three times with wash buffer, with each wash lasting 5 minutes. One drop (30 µl) of DAB Chromogen Concentrate was added to 1 mL of DAB Diluent and mixed thoroughly before use. Then, 100–400 µl of Signal Stain DAB was applied to each section and monitored closely for 1–10 minutes until an acceptable staining intensity was achieved. Finally, the slides are rinsed in deionized water, followed by counterstaining with hematoxylin. The sections are then washed twice in deionized water for 5 minutes each. In the stained sections, the cell nuclei appear blue, while the antibody stained with the DAB chromogen provides a brown contrast, enhancing the visualization of tissue morphology. Before mounting the coverslip, the sections must be rehydrated. To rehydrate the tissue sections, they are placed in two containers of 95% ethanol for 10 seconds each, followed by two containers of 100% ethanol for 10 seconds each, and two containers of xylene for 10 seconds each. The sections are then mounted with coverslips using mounting medium, ensuring no air bubbles are introduced. Finally, the tissue sections are examined under a light microscope. The evaluation of immune-staining was performed by pathologists who was blind to experiment details, and the H -score was calculated according to^136,137^. The immune-positive cells in each region of interest (ROI) were counted using a counting grid, and their proportion relative to the total counterstained cell population was calculated. The stained areas within the ROI were digitally marked, and the percentage of stained areas was determined using a computer program. Protein expression intensity was evaluated using the immune-reactive score (IRS) for interpreting IHC data. The IRS ranges from 0 to 12, calculated by multiplying the proportion score of positive cells (0–4) with the staining intensity score (0–3).

The microscopic examination of immune-stained sections was examined. *Images are captured by LABOMED Trinocular inverted phase contrast microscope model TCM400 microscope and the Atlas 16MP Cmos USB Camera software (LABOMED, USA). The magnification power is 40x, scale bar: 50µm.* Adopted immune-reactive score (IRS) was proceeded as following:

| **A (percentage of positive cells)** | **B (intensity of staining)** | **IRS score (multiplication of A and B)** |
| --- | --- | --- |
| **0 = no positive cells** | 0 = no color reaction | 0-1 = negative |
| **1 = <10% of positive cells** | 1 = mild reaction | 2-3 = mild |
| **2 = 11-50% positive cells** | 2 = moderate reaction | 4-8 = moderate |
| **3 = 51-80% positive cells** | 3 = intense reaction | 9-12 = strongly positive |
| **4 = >80% positive cells** | **Final IRS score (A × B): 0-12** | |

**References:**

1. Elmaaty, A. A. *et al.* In Silico and In Vitro Studies for Benzimidazole Anthelmintics Repurposing as VEGFR-2 Antagonists: Novel Mebendazole-Loaded Mixed Micelles with Enhanced Dissolution and Anticancer Activity. *ACS Omega* **7**, 875-899, doi:10.1021/acsomega.1c05519 (2022).
2. Saleh, A. H. *et al.* Deciphering the molecular basis of the kappa opioid receptor selectivity: A Molecular Dynamics study. *Journal of Molecular Graphics and Modelling* **106**, 107940, doi:10.1016/j.jmgm.2021.107940 (2021).
3. Ross, G. A., Rustenburg, A. S., Grinaway, P. B., Fass, J. & Chodera, J. D. Biomolecular Simulations under Realistic Macroscopic Salt Conditions. *The journal of physical chemistry. B* **122**, 5466-5486, doi:10.1021/acs.jpcb.7b11734 (2018).
4. Zaki, A. A., Ashour, A., Elhady, S. S., Darwish, K. M. & Al-Karmalawy, A. A. Calendulaglycoside A showing potential activity against SARS-CoV-2 main protease: Molecular docking, molecular dynamics, and SAR studies. *Journal of traditional and complementary medicine* **12**, 16-34, doi:10.1016/j.jtcme.2021.05.001 (2022).
5. Tuble, S. C., Anwar, J. & Gale, J. D. An Approach to Developing a Force Field for Molecular Simulation of Martensitic Phase Transitions between Phases with Subtle Differences in Energy and Structure. *J. Am. Chem. Soc.* **126**, 396-405, doi:10.1021/ja0356131 (2004).
6. Darden, T., York, D. & Pedersen, L. Particle mesh Ewald: An N⋅log(N) method for Ewald sums in large systems. *J. Chem. Phys.* **98**, 10089-10092, doi:10.1063/1.464397 (1993).
7. Hess, B., Bekker, H., Berendsen, H. J. C. & Fraaije, J. G. E. M. LINCS: A linear constraint solver for molecular simulations. *J. Comput. Chem.* **18**, 1463-1472, doi:10.1002/(SICI)1096-987X(199709)18:12<1463::AID-JCC4>3.0.CO;2-H (1997).
8. Páll, S. & Hess, B. A flexible algorithm for calculating pair interactions on SIMD architectures. *Comput. Phys. Comm.* **184**, 2641-2650, doi:<https://doi.org/10.1016/j.cpc.2013.06.003> (2013).
9. Kumari, R., Kumar, R. & Lynn, A. g_mmpbsa—A GROMACS Tool for High-Throughput MM-PBSA Calculations. *Journal of Chemical Information and Modeling* **54**, 1951-1962, doi:10.1021/ci500020m (2014).
10. Nafie, M. S. *et al.* Control of ER-positive breast cancer by ERα expression inhibition, apoptosis induction, cell cycle arrest using semisynthetic isoeugenol derivatives. *Chemico-Biological Interactions* **351**, 109753 (2022).
11. Nafie, M. S. & Boraei, A. T. Exploration of novel VEGFR2 tyrosine kinase inhibitors via design and synthesis of new alkylated indolyl-triazole Schiff bases for targeting breast cancer. *Bioorganic Chemistry* **122**, 105708 (2022).
12. Abd El-Meguid, E. A., Naglah, A. M., Moustafa, G. O., Awad, H. M. & El Kerdawy, A. M. Novel benzothiazole-based dual VEGFR-2/EGFR inhibitors targeting breast and liver cancers: Synthesis, cytotoxic activity, QSAR and molecular docking studies. *Bioorganic & Medicinal Chemistry Letters* **58**, 128529 (2022).
